# Selective Modification of Streptozotocin at the C3 Position to Improve Its Bioactivity as Antibiotic and Reduce Its Cytotoxicity towards Insulin-Producing β Cells

**DOI:** 10.3390/antibiotics9040182

**Published:** 2020-04-15

**Authors:** Ji Zhang, Liubov Yakovlieva, Bart J. de Haan, Paul de Vos, Adriaan J. Minnaard, Martin D. Witte, Marthe T. C. Walvoort

**Affiliations:** 1 Stratingh Institute for Chemistry, University of Groningen, Nijenborgh 7, 9747 AG Groningen, The Netherlands; zhangji1988614@hotmail.com (J.Z.); l.yakovlieva@rug.nl (L.Y.); a.j.minnaard@rug.nl (A.J.M.); m.d.witte@rug.nl (M.D.W.); 2 Department of Pathology and Medical Biology, University of Groningen Medical Center, Hanzeplein 1, 9700 RB Groningen, The Netherlands; b.j.de.haan@umcg.nl (B.J.d.H.); p.de.vos@umcg.nl (P.d.V.)

**Keywords:** antibiotics, regioselective oxidation, streptozotocin, β cells

## Abstract

With the increasing resistance of bacteria to current antibiotics, novel compounds are urgently needed to treat bacterial infections. Streptozotocin (STZ) is a natural product that has broad-spectrum antibiotic activity, albeit with limited use because of its toxicity to pancreatic β cells. In an attempt to derivatize STZ through structural modification at the C3 position, we performed the synthesis of three novel STZ analogues by making use of our recently developed regioselective oxidation protocol. Keto-STZ (**2**) shows the highest inhibition of bacterial growth (minimum inhibitory concentration (MIC) and viability assays), but is also the most cytotoxic compound. Pre-sensitizing the bacteria with GlcNAc increased the antimicrobial effect, but did not result in complete killing. Interestingly, *allo*-STZ (**3**) revealed moderate concentration-dependent antimicrobial activity and no cytotoxicity towards β cells, and deoxy-STZ (**4**) showed no activity at all.

## 1. Introduction

The most applied therapeutics for the management of bacterial infections are still antibiotics. However, an increasing number of bacteria develops resistance against current treatments, and novel substances and methods to combat bacterial infection are therefore needed. Next to developing completely new compounds with a different mode of action, the promising strategy of modifying currently available antibiotics has gained attention. Derivatization may overcome the existing resistance towards the antibiotic, can reduce its toxicity, and may even result in analogues that inhibit bacterial growth via a new mode of action. The effectiveness of this approach has been illustrated by the synthesis of derivatives of vancomycin, a potent inhibitor of bacterial cell wall synthesis, that overcome vancomycin-resistant enterococci infections [1]. Major efforts have been directed towards the (semi) synthesis of analogues of carbohydrate-based antibiotics, and in particular aminoglycosides [2,3]. Aminoglycosides are natural products that are highly cationic in nature and show bactericidal activity, as they bind the 30S component of the bacterial ribosome, thereby blocking protein synthesis and inhibiting bacterial growth [4]. The adverse effects of aminoglycosides include renal toxicity, and ototoxicity at high concentrations. In addition, there are multiple enzymes that work on aminoglycosides to inactivate their antibiotic activity, which limits their use in the clinic [5]. A large variety of structural analogues, including amphiphilic aminoglycosides [6], conformationally-locked aminoglycosides, and glycosylated aminoglycosides [7,8] have been synthesized to overcome the adverse effects [3]. These efforts led to the discovery of derivatives that cannot be modified by aminoglycoside-inactivating enzymes [9,10], that have reduced toxicity [6], and even inhibit bacterial growth via new mechanisms of action [11,12].

**Figure 1:**
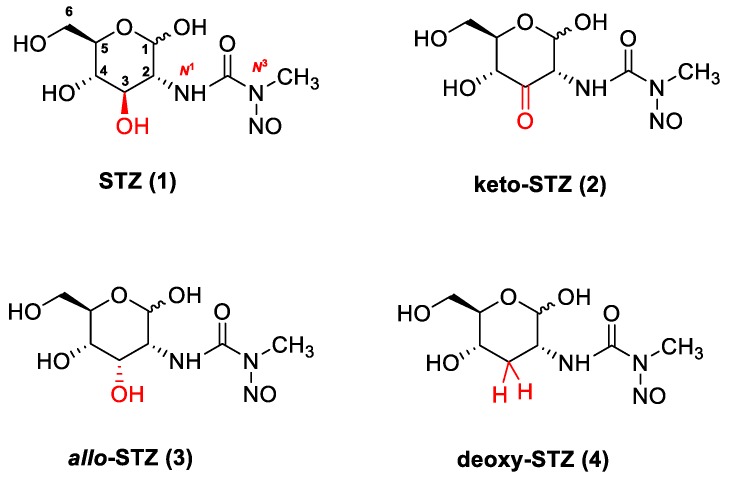
Structures of Streptozotocin (STZ) (**1**), and the STZ-derivatives (**2**–**4)** synthesized and evaluated in this project.

In our ongoing research program, we have been developing site-selective methods to functionalize and modify carbohydrates without using chemical protecting groups. We reasoned that these methods could give straightforward access to new derivatives of carbohydrate-based antibiotics. In this paper, we describe our efforts on modifying streptozotocin (CAS No. 1883-66-4, STZ, **1**) as a starting point for chemical derivatization to maintain antimicrobial activity but reduce the side effects such as toxicity towards insulin-producing β cells. STZ (2-deoxy-2-(3-methyl-3-nitrosourea)-1-d-glucopyranose) is a derivative of *N*-acetylglucosamine produced by *Streptomyces achromogenes* var. *streptozoticus*, and its chemical structure is depicted in Figure 1. STZ was first discovered and characterized in the 1960s and exists as an equal mixture of its α and β anomer [13,14]. STZ is most stable at pH 4.5 and degrades rapidly in alkaline solutions [15]. It was shown to exhibit broad-spectrum antibacterial activity and possesses antitumor and mutagenic properties [16]. Although initially embraced as an antibiotic, also because of its apparent lack of toxicity to mammalian cells, STZ turned out to be very toxic to the insulin-producing β cells of the pancreas [13]. As a result of this cytotoxicity, the application of STZ as an antibiotic was terminated, and **1** is nowadays mostly used to generate experimental animal models of diabetes [16]. 

STZ is specifically toxic to pancreatic β cells that secrete insulin, because it is efficiently transported into the β cells by the glucose transport protein 2 (GLUT2) [17,18]. This may be explained by the structural similarities to glucose, as STZ is a 2-amino-2-deoxyglucoside that appears to act as a carrier for the *N*^3^-methyl-*N*^3^-nitrosourea group [19]. In contrast to glucose itself, STZ is not recognized by other glucose transporters. Since GLUT2 is primarily found in the cellular membranes of insulin-producing β cells, STZ is especially toxic to the pancreas. It is now also approved for use in islet-cell carcinomas and malignant carcinoid tumors in humans [20]. 

In bacteria, STZ is imported through the phosphoenolpyruvate-carbohydrate phosphotransferase system (PTS) [21], and accumulates intracellularly in its phosphorylated form [22]. The antibacterial activity arises from the release of diazomethane through the hydrolysis of STZ, leading to DNA damage. Already at low intracellular concentrations, septum formation in the bacteria is shown to be affected, the bacteria grow into filaments, and the surviving cells are also mutagenized [23]. Next to antibacterial and diabetogenic activity, STZ has also been reported to display anti-leukemic and mutagenic activity [16]. 

Because of the different biological activities observed with STZ, research has been devoted to understand the relation between structural modifications of STZ and the resulting biological effects. Fully acetylated streptozotocin has no antibacterial activity, but shows an increased inhibition of the growth of L-1210 leukemia cells compared to STZ [24]. Furthermore, replacement of the methyl group at *N*^3^ of the nitrosourea by an ethyl or *n*-butyl substituent results in the loss of antibacterial activity. Inversion of the 4-hydroxyl group of streptozotocin (from *gluco* to *galacto* configuration) leads to the loss of antibacterial activity against *Proteus vulgaris*. Inversion of the C-2 hydroxyl (from *gluco* to *manno* configuration) reduced the antibacterial activity markedly as well [24]. O-alkylation of the anomeric hydroxyl of STZ improves the antitumor activities and reduces toxicity, but leads to a loss of the antibacterial and diabetogenic activity. [25] In addition, methylation of the anomeric hydroxyl group of STZ eliminates antibacterial activity, and α-methyl STZ is twice as active as β-methyl STZ against cultures of leukemia L1210. The cytotoxicity of β-methyl STZ is identical to STZ [24].

So far, in the reported modifications of STZ, the C3-OH position has not been altered (Figure 1). To complement the reported modifications of STZ, we decided to focus our efforts on the C3-OH position. The targeted analogues are depicted in Figure 1, and include the C3-keto produced by oxidation (**2**), the C3-*allo*-OH produced by inversion (**3**), and the C3-deoxy produced by reduction (**4**). Ultimately, we were aiming to retain or improve the antibacterial activity of STZ, while at the same time reducing the cytotoxicity to β cells. We hypothesized that changing the configuration at the C3 position may reduce the affinity of the STZ-analogue to GLUT2, resulting in reduced toxicity to β cells. At the same time, because bacteria express unique transport systems that are absent in mammalian cells, such as a d-allose transporter [26] and the PTS system, the altered STZ compounds may still be imported by bacteria, resulting in antibacterial activity. Here we present the chemical synthesis of STZ analogues **2**–**4**, and the biological evaluation using minimum inhibitory concentration (MIC) and growth-viability assays on *E. coli* K12 cells as well as cytotoxicity studies on insulin-producing β cells.

## 2. Results

### 2.1. Chemical Synthesis of STZ Derivatives ***2**–**4***

**Scheme 1:**
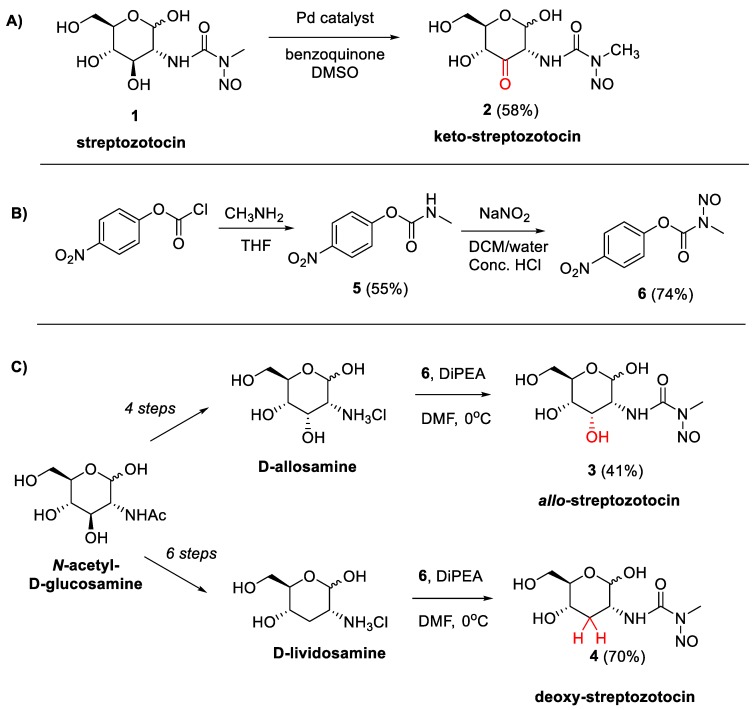
(**A**) One-step preparation of keto-STZ (**2**); (**B**) Synthesis of the *N*-nitrosocarbamate **6**; (**C**) Synthesis of analogues **3** and **4** starting from *N*-acetyl-d-glucosamine using late-stage methyl nitrosourea introduction to d-allosamine and d-lividosamine [32].

The chemical synthesis of the three STZ derivatives exploited the regioselective oxidation protocol developed in our group, as published previously [27,28] In short, largely unprotected carbohydrates are treated with [(neocuproine)Pd(μ-OAc)]_2_(OTf)_2_ as the catalyst and benzoquinone as the oxidant to selectively oxidize the C3-OH to the keto-functionality. Gratifyingly, this procedure could be employed to oxidize STZ directly, yielding keto-STZ (**2**) in one step and decent yield (58%) after purification (Scheme 1A). For the synthesis of the analogues **3** and **4**, the *N*-nitroso moiety was introduced in the final stage using *N*-nitrosocarbamate reagent **6** [29,30,31]. As depicted in Scheme 1B, 4-nitrophenyl *N*-nitroso-*N*-methylcarbamate **6** was prepared by treating 4-nitrophenyl chloroformate in THF at 0 °C with a solution of methylamine in THF to afford 4-nitrophenyl methylcarbamate **5**. Nitrosation of **5** with NaNO_2_ was carried out in a mixture of DCM and 12 M HCl in water and produced compound **6**. d-Allosamine and d-lividosamine were prepared using reported procedures [32], and treated with reagent **6** to obtain *allo*-STZ (**3**, 41%) and deoxy-STZ (**4**, 70%) (Scheme 1C). *Allo*-STZ and deoxy-STZ were isolated as a mixture of the pyranose and furanose forms, with the pyranose form being the major component, and fully characterized. STZ and its analogues are best stored as solids at −20 °C and shielded from light. Solutions of STZ are most stable at pH 4.5, but are known to slowly release NO gas at ambient temperature [33]. Since NO release cannot be prevented even at −80 °C, solutions are prepared immediately before use. 

### 2.2. Biological Evaluation of STZ Compounds ***1**–**4***

**Figure 2:**
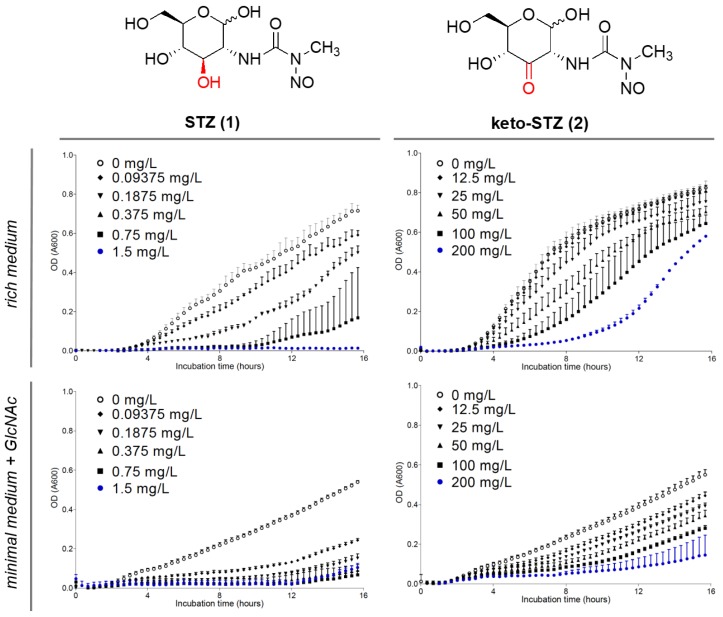
Growth curves of *E. coli* to determine the MIC value for STZ **1** (**top left**) and keto-STZ **2** (**top right**) in rich medium, and for STZ **1** (**bottom left**) and keto-STZ **2** (**bottom right**) in minimal medium + GlcNAc. Experiments are performed in triplicate.

Next, we started the biological evaluation of compounds **1**–**4** with a standard MIC assay, where the compounds are incubated with *E. coli* K12 (TOP10) cells for 16 h. As depicted in Table 1 and Figure 2, the parent STZ compound **1** exhibited potent antimicrobial activity as expected, with an MIC value of 1.5 mg/L in rich medium (lysogeny broth, LB). This corresponds with reported values, which lie between 0.4 and 100 mg/L depending on the bacteria and even the *E. coli* strain [23]. Interestingly, when the cells were grown in minimal medium with GlcNAc as the sole carbon source prior to addition of STZ **1** in minimal medium containing glycerol as the carbon source, incomplete killing was observed with the same concentrations (MIC > 1.5 mg/L). When the novel STZ-analogues **2**–**4** were evaluated, a large reduction in antimicrobial activity was observed. Only minor impairment of bacterial growth was observed for treatment with analogues **2**–**4** for 16 h (MIC values > 200 mg/L, Table 1). However, when the MIC studies were performed with continuous monitoring of bacterial growth during the overnight incubation with STZ **1** or analogues **2**–**4**, a more detailed picture of the inhibition profile was obtained, revealing significant differences in growth behavior. Overall, the growth rate in minimal medium was reduced as compared to growth in rich medium. Parent STZ (**1**), keto-STZ (**2**) and *allo*-STZ (**3**) all revealed concentration-dependent inhibition that was increased after pre-sensitizing the bacteria with GlcNAc (Figure 2 and Supplementary Figure S1). At the same time, pre-sensitizing led to an increase in MIC value for STZ (**1**), as even the highest concentration of STZ (1.5 mg/L) showed outgrowth of cells after 12 h. Interestingly, pre-sensitizing with ribose (a configurational homolog of allose) increased the susceptibility of *E. coli* to *allo*-STZ (**3**) in a similar way, while the effect of deoxy-STZ (**4**) was unaffected (Supplementary Figure S1).

**Figure 3:**
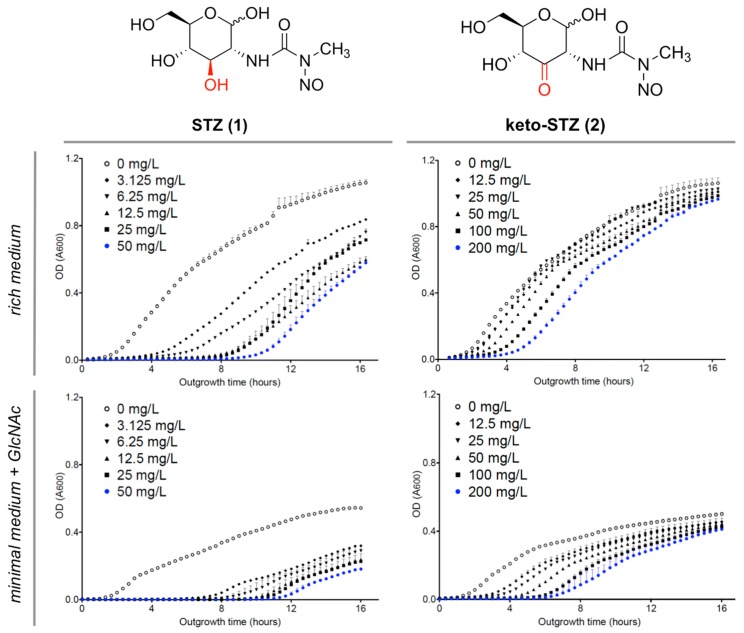
Growth-based viability curves of *E. coli* for STZ **1** (**top left**) and keto-STZ **2** (**top right**) in rich medium, and for STZ **1** (**bottom left**) and keto-STZ **2** (**bottom right**) in minimal medium + GlcNAc. Experiments are performed in triplicate.

An alternative way to asses antimicrobial activity is to identify the percentage of viable bacteria after a short treatment with the antibiotic (2 h). In this so-called growth-based viability assay, originally developed by Haynes and co-workers for the toxicity screening of nanomaterials [34], the percentage of viable cells is determined by correlating the growth (OD_600_) of the treated samples to control samples with a known number of cells that are grown under the same conditions. The advantage of using relative viabilities is that it allows straightforward comparison of viability, even when the experiments have been performed under different growth conditions. The results of the growth-based viability assay are presented in Table 2. In rich-medium conditions, STZ (**1**) showed the largest effect on bacterial growth, with a viability of 0.0035% after treatment with 50 mg/L. Of the STZ-derivatives, keto-STZ (**2**) was the most active inhibitor of growth (8% of viability with 200 mg/L), whereas *allo*-STZ (**3**) showed a remaining viability of 29%. Deoxy-STZ (**4**) showed no significant delay in growth, resulting at a calculated viability of 127%, even at the highest concentration tested (200 mg/L). These results corroborate those of the MIC assays. When the viability assays were performed on cells pre-sensitized with GlcNAc, a similar trend was observed as compared to the MIC assays (Figure 3). The activity of STZ (**1**) was increased two-fold, with a resulting viability of 0.0019% after treatment with 50 mg/L. Keto-STZ (**2**) also showed higher activity in the same concentration range compared to its effect on non-presensitized cells (0.1% viable cells with 200 mg/L). In contrast, the antibacterial activity of *allo*-STZ (**3**) remained the same, and deoxy-STZ (**4**) showed no inhibitory effect on bacterial growth, also after pre-sensitizing with ribose (Supplementary Figure S2).

**Figure 4:**
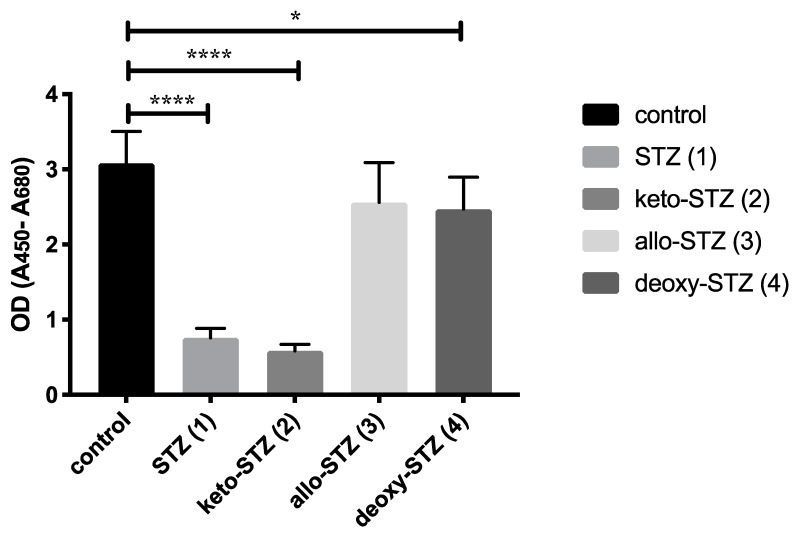
Effect of compounds **1**–**4** (5 mM) on viability of β cells. Significant differences compared to the control were indicated by * *p* < 0.05 or **** *p* < 0.0001.

From the antimicrobial assays above it is clear that the parent STZ (**1**) remains the most active in inhibiting bacterial growth, while keto-STZ (**2**) and allo-STZ (**3**) showed moderate to poor activity. As an important goal of this project is to understand the structural motifs important for antimicrobial and cytotoxic activities, we explored the toxicity of compounds **1**–**4** on insulin-producing β cells. Using the WST-1 dye as a colorimetric read-out of cell proliferation and mitochondrial activity, the effect of compounds **1**–**4** was measured, as depicted in Figure 4. Compared to the negative inhibition control (incubation with citrate), STZ (**1**) shows a large reduction in β cell viability. Unfortunately, this result is mimicked by keto-STZ (**2**), which shows a similarly high cytotoxicity. In contrast, *allo*-STZ (**3**) and deoxy-STZ (**4**) show a negligible amount of cytotoxicity in this assay.

## 3. Discussion

To overcome the increasing resistance of bacteria to current antibiotics, novel antimicrobial compounds are desperately needed. Using our regioselective oxidation method, we generated three novel STZ analogues. It was exciting to see that keto-derivative **2** could be accessed in good yields by oxidizing STZ **1** directly. The robust way of *N*-nitrosourea introduction using *N*-nitrosocarbamate reagent **6** proved useful for the generation of the STZ analogues based on d-allosamine (*allo*-STZ, **3**) and d-lividosamine (deoxy-STZ, **4**). It is clear from the MIC assays that the parent STZ compound (**1**) displayed the largest antimicrobial activity on *E. coli* K12 (TOP10) cells, with only 1.5 mg/L needed for complete killing. When the *E. coli* cells were first treated with GlcNAc before incubation with STZ, the impact of STZ was reduced. Earlier studies already revealed that the effect of exogenous GlcNAc on carbohydrate metabolism and bacterial growth is complex [23]. When GlcNAc and STZ were added simultaneously, *E. coli* cells have been observed to import less STZ, suggesting that they compete for the same transport machinery, presumably the PTS system [22]. In contrast, when the cells are first pre-sensitized with GlcNAc, the upregulation of transport machinery is suggested to result in a higher sensitivity to STZ **1** and keto-STZ **2** [35]. The latter effect is especially observed in the first 8 h of incubation (Figure 2), where the growth rate is significantly impacted by the lower concentrations compared to rich medium (0.1875 and 0.375 mg/L for STZ, 12.5 and 25 mg/L for keto-STZ). This may reflect the upregulation of transport machinery in the presence of GlcNAc, which primes the bacterial cells to increase the uptake of STZ, resulting in an increased sensitivity. However, after 8 h the OD_600_ increases again and reveals incomplete killing even with the highest concentrations of STZ **1** (Figure 2). This result may reflect a loss of the sensitization effect over time, and a reduction of the expression of GlcNAc transport machinery. Alternatively, STZ and analogues may be slowly degrading under the assay conditions [33]. The growth-based viability experiments produce a similar trend (Figure 3), as the largest delay in outgrowth was observed for parent STZ (**1**) and keto-STZ (**2**). It is interesting to note that pre-sensitizing with ribose increased the susceptibility of *E coli* cells to *allo*-STZ (**3**), but not to deoxy-STZ (**4**). This may indicate the upregulation of the d-allose operon, as described before [26]. Unfortunately, the trend of antibacterial activity of compounds **1**–**4** is mirrored in the cytotoxicity experiment. When incubated with insulin-producing β cells, compounds **1** and **2** show the highest reduction in cell density (Figure 4), whereas compounds **3** and **4** are only marginally toxic to the cells. We postulate that the activity of keto-STZ (**2**) in both the antimicrobial activity and cytotoxicity assays may be explained by the hydrate, which results after addition of water to the ketone functionality. Even though the hydrate has not been observed in NMR, as hydration at keto-C3 (^13^C NMR~204 ppm for the keto) would result in a significant upfield shift (^13^C NMR~95 ppm for the hydrate) [36] that was not observed, a small amount of the keto may be hydrated in situ. When the hydrate is formed on the C3 position, the resulting structure may mimic the configuration of STZ more closely. The moderate but concentration-dependent antimicrobial activity of *allo*-STZ (**3**) is an exciting result, because this STZ analogue did not show significant cytotoxicity to β cells.

In conclusion, we have described a straightforward and robust method to derivatize STZ on the C3 position, and generated three novel analogues by oxidation, inversion and reduction. This late-stage modification of largely unprotected carbohydrates, compatible with sensitive functional groups such as a free anomeric hydroxyl group and a reactive *N*-nitroso functionality, allows straightforward access to analogues that normally require full syntheses. We foresee that this late stage modification approach can be expanded, at least to other carbohydrate-based drugs, like the many other aminoglycoside antibiotics. In the same vein it is obvious that not only epimerization and hydroxy group removal are possible but also conversion of the keto function into other functional groups and handles. Of the prepared analogues, keto-STZ (**2**) showed the highest antimicrobial activity and largest delay in bacterial growth, albeit significantly lower than parent STZ (**1**). Moreover, the toxicity to β cells was similarly largest for keto-STZ (**2**), limiting the applicability of this compound as an antibiotic. Although the *allo*-STZ (**3**) analogue revealed a promising selectivity for the killing of bacterial cells over pancreatic β cells, the antimicrobial activity is not competitive with STZ. This suggests that the bacterial carbohydrate uptake systems only moderately tolerate the modification of the C3 position in glucose and GlcNAc analogues. This research invites a more thorough analysis of the various carbohydrate uptake mechanisms bacteria use. Once we know the precise binding of glucose and GlcNAc, also with respect to the anomeric configuration, and the pyranose versus furanose form, rational design and synthesis of STZ analogues will be possible. Alternatively, chemical modifications can be investigated to stimulate the bacterial uptake of STZ analogues.

## 4. Materials and Methods

### 4.1. Determination of MIC Values

The minimum inhibitory concentration (MIC) of STZ derivatives **2**–**4**, in comparison to STZ (**1**), was determined according to the recommendations proposed by the Clinical and Laboratory Standard Institute (CLSI) [37], using LB as rich medium. See the Supplementary Information for a detailed procedure.

### 4.2. Growth-Based Viability Assays

To better assess the impact of the STZ derivatives **2**–**4** on bacterial growth and viability, growth-based viability assays were performed [34]. The assay was performed in rich medium (lysogeny broth, LB) as well as in minimal medium with *N*-acetylglucosamine (GlcNAc) or ribose as additives to study the effect of these compounds on the uptake of the streptozotocin-analogues. Bacterial cells (*Escherichia coli* K12 TOP10) were first exposed to the streptozotocin derivatives (either in rich or minimal medium) at room temperature and constant shaking to ensure good mixing. Subsequently, a portion of the exposed mixture was diluted 40× by addition to fresh medium to alleviate the effect of the antibiotic, and the cells were incubated in a Biotek plate reader for 16 h at 37 °C with optical density (OD) measurements at 600 nm taking place every 20 min preceded by 30 s of shaking. The resulting data was processed to obtain viability values, as described in detail in the Supplementary Information.

### 4.3. Cytotoxicity Assays

The MIN6 cell line (pancreatic β cells) was purchased from American Type Culture Collection (ATCC, Manassas, VA, USA). MIN6 cells (passages 30–45) were cultured in DMEM High glucose medium (Lonza, Basal, Switzerland), containing 15% fetal bovine serum (FBS, Lonza), 50 μmol/L β-mercaptoethanol, 2 mmol/L l-glutamine, 50 U/mL penicillin, and 50 μg/mL streptomycin (all from Sigma-Aldrich, St. Louis, MO, USA). Cells were cultured at 37 °C in a humidified atmosphere containing 95% air and 5% CO_2_. The effect of streptozotocin (STZ) and the derivatives on β cell viability was determined by the cell proliferation reagent WST-1 (Roche, Indianapolis, USA). Briefly, MIN6 cells (1 × 10^5^ cells/well) were seeded in 96-well plates. Cells were cultured overnight and the following day incubated with or without STZ or analogue (Sigma-Aldrich) at 5 mM for 48 and 72 h followed by WST-1 assay. After 30 min incubation with WST-1 (10 μL/well) at 37 °C, the absorbance was measured at 450 nm using a Bio-Rad Benchmark Plus microplate spectrophotometer reader (Bio-Rad Laboratories B.V, Veenendaal, The Netherlands.

## Figures and Tables

**Table 1:** Results from the minimum inhibitory concentration (MIC) assays using compounds **1**–**4**.

Compound	MIC Value (mg/L)		
	LB ^a^	MM ^b^ + GlcNAc	MM ^b^ + Ribose
STZ (**1**)	1.5	>1.5	n.d.
keto-STZ (**2**)	>200	>200	n.d.
*allo*-STZ (**3**)	>200	>200	>200
deoxy-STZ (**4**)	>200	>200	>200

^a^ LB = rich medium; ^b^ MM = minimal medium.

**Table 2:** Results from the growth-based viability assays using compounds **1**–**4**.

Compound	Viability		
	LB ^a^	MM ^b^ + GlcNAc	MM ^b^ + Ribose
STZ (**1**) (50 mg/L)	0.0035%	0.0019%	n.d.
keto-STZ (**2**) (200 mg/L)	8%	0.1%	n.d.
*allo*-STZ (**3**) (200 mg/L)	29%	29%	21%
deoxy-STZ (**4**) (200 mg/L)	127%	170%	202%

^a^ LB = rich medium; ^b^ MM = minimal medium.

## Supplementary Materials

The following are available online at https://www.mdpi.com/2079-6382/9/4/182/s1, Figure S1: Growth curves of *E. coli* TOP10 to determine the MIC value for *allo*-STZ **3** (top left) and deoxy-STZ **4** (top right) in rich medium, *allo*-STZ **3** (middle left) and deoxy-STZ **4** (middle right) in minimal medium + GlcNAc, and *allo*-STZ **3** (bottom left) and deoxy-STZ **4** (bottom right) in minimal medium + ribose. Experiments are performed in triplicate; Figure S2: Growth-based viability curves of *E. coli* TOP10 for *allo*-STZ **3** (top left) and deoxy-STZ **4** (top right) in rich medium, *allo*-STZ **3** (middle left) and deoxy-STZ **4** (middle right) in minimal medium + GlcNAc, and *allo*-STZ **3** (bottom left) and deoxy-STZ **4** (bottom right) in minimal medium + ribose. Experiments are performed in duplicate.

## References

[1] Mühlberg E., Umstätter F., Kleist C., Domhan C., Mier W., Uhl P. (2020). Renaissance of vancomycin: Approaches for breaking antibiotic resistance in multidrug-resistant bacteria. Can. J. Microbiol..

[2] Khan F., Pham D.T.N., Kim Y.-M. (2020). Alternative strategies for the application of aminoglycoside antibiotics against the biofilm-forming human pathogenic bacteria. Appl. Microbiol. Biotechnol..

[3] Chandrika N.T., Garneau-Tsodikova S. (2018). Comprehensive review of chemical strategies for the preparation of new aminoglycosides and their biological activities. Chem. Soc. Rev..

[4] Kotra L.P., Haddad J., Mobashery S. (2000). Aminoglycosides: Perspectives on Mechanisms of Action and Resistance and Strategies to Counter Resistance. Antimicrob. Agents Chemother..

[5] Ramirez M.S., Tolmasky M.E. (2010). Aminoglycoside modifying enzymes. Drug Resist. Updat..

[6] Kato T., Yang G., Teo Y., Juskeviciene R., Perez-Fernandez D., Shinde H.M., Salian S., Bernet B., Vasella A., Böttger E.C. (2015). Synthesis and Antiribosomal Activities of 4′-O-, 6′-O-, 4″-O-, 4′,6′-O- and 4″,6″-O-Derivatives in the Kanamycin Series Indicate Differing Target Selectivity Patterns between the 4,5- and 4,6-Series of Disubstituted 2-Deoxystreptamine Aminoglycoside Antibiotics. ACS Infect. Dis..

[7] Chen W., Matsushita T., Shcherbakov D., Boukari H., Vasella A., Böttger E.C., Crich D. (2014). Synthesis, antiribosomal and antibacterial activity of 4′-O-glycopyranosyl paromomycin aminoglycoside antibiotics. MedChemComm.

[8] Matsushita T., Chen W., Juskeviciene R., Teo Y., Shcherbakov D., Vasella A., Böttger E.C., Crich D. (2015). Influence of 4′-O-Glycoside Constitution and Configuration on Ribosomal Selectivity of Paromomycin. J. Am. Chem. Soc..

[9] Sati G.C., Sarpe V.A., Furukawa T., Mondal S., Mantovani M., Hobbie S.N., Vasella A., Böttger E.C., Crich D., Mantovani M. (2019). Modification at the 2′-Position of the 4,5-Series of 2-Deoxystreptamine Aminoglycoside Antibiotics To Resist Aminoglycoside Modifying Enzymes and Increase Ribosomal Target Selectivity. ACS Infect. Dis..

[10] Sonousi A., Sarpe V.A., Brilkova M., Schacht J., Vasella A., Böttger E.C., Crich D. (2018). Effects of the 1-N-(4-Amino-2S-hydroxybutyryl) and 6′-N-(2-Hydroxyethyl) Substituents on Ribosomal Selectivity, Cochleotoxicity, and Antibacterial Activity in the Sisomicin Class of Aminoglycoside Antibiotics. ACS Infect. Dis..

[11] Herzog I.M., Feldman M., Eldar-Boock A., Satchi-Fainaro R., Fridman M. (2013). Design of membrane targeting tobramycin-based cationic amphiphiles with reduced hemolytic activity. MedChemComm.

[12] Fosso M.Y., Zhu H., Green K.D., Garneau-Tsodikova S., Fredrick K. (2015). Tobramycin variants with enhanced ribosome-targeting activity. ChemBioChem.

[13] Ogbonnaya E.C., Eleazu K., Chukwuma S., Essien U.N. (2013). Review of the mechanism of cell death resulting from streptozotocin challenge in experimental animals, its practical use and potential risk to humans. J. Diabetes Metab. Disord..

[14] Agarwal M. (1980). Streptozotocin: Mechanisms of action—Proceedings of a Workshop Held on 21 June 1980, Washington, Dc. FEBS Lett..

[15] Reusser F. (1971). Mode of Action of Streptozotocin. J. Bacteriol..

[16] King A.J.F. (2012). The use of animal models in diabetes research. Br. J. Pharmacol..

[17] Wang Z., Gleichmann H. (1998). GLUT2 in pancreatic islets: Crucial target molecule in diabetes induced with multiple low doses of streptozotocin in mice. Diabetes.

[18] Schnedl W.J., Ferber S., Johnson J.H., Newgard C.B. (1994). STZ transport and cytotoxicity. Specific enhancement in GLUT2-expressing cells. Diabetes.

[19] Elsner M., Guldbakke B., Tiedge M., Munday R., Lenzen S. (2000). Relative importance of transport and alkylation for pancreatic beta-cell toxicity of streptozotocin. Diabetol..

[20] Moertel C.G., Hanley J.A., Johnson L.A. (1980). Streptozocin Alone Compared with Streptozocin plus Fluorouracil in the Treatment of Advanced Islet-Cell Carcinoma. N. Engl. J. Med..

[21] Postma P.W., Lengeler J.W., Jacobson G.R. (1993). Phosphoenolpyruvate:carbohydrate phosphotransferase systems of bacteria. Microbiol. Rev..

[22] Ammer J., Brennenstuhl M., Schindler P., Holtje J.V., Zahner H. (1979). Phosphorylation of streptozotocin during uptake via the phosphoenolpyruvate: Sugar phosphotransferase system in Escherichia coli. Antimicrob. Agents Chemother..

[23] Lengeler J. (1980). Analysis of the physiological effects of the antibiotic streptozotocin on Escherichia coli K 12 and other sensitive bacteria. Arch. Microbiol..

[24] Bannister B. (1972). Synthesis and Biological-Activities of some Analogs of Streptozotocin. J. Antibiot..

[25] Iwasaki M., Ueno M., Ninomiya K., Sekine J., Nagamatsu Y., Kimura G. (1976). Alkyl streptozotocin analogues with improved biological activities. J. Med. Chem..

[26] Kim C., Song S., Park C. (1997). The D-allose operon of Escherichia coli K-12. J. Bacteriol..

[27] Jäger M., Hartmann M., De Vries J.G., Minnaard A.J. (2013). Catalytic Regioselective Oxidation of Glycosides. Angew. Chem. Int. Ed..

[28] Jumde V.R., Eisink N.N.H.M., Witte M.D., Minnaard A.J. (2016). C3 Epimerization of Glucose, via Regioselective Oxidation and Reduction. J. Org. Chem..

[29] Martinez J., Oiry J., Imbach J.L., Winternitz F. (1980). A Selective Synthesis of N-Alkyl N-Nitrosoureas. Eur. J. Med. Chem..

[30] Martinez J., Oiry J., Imbach J.L., Winternitz F. (1982). Activated N-nitrosocarbamates for regioselective synthesis of N-nitrosoureas. J. Med. Chem..

[31] Gassmann N., Stoos F., Meier A., Helali S.E., Hardegger E. (1975). Varianten im Zuckerteil des Streptozotocins. Helv. Chim. Acta.

[32] Zhang J., Eisink N.N.H.M., Witte M.D., Minnaard A.J. (2018). Regioselective Manipulation of GlcNAc Provides Allosamine, Lividosamine, and Related Compounds. J. Org. Chem..

[33] Goud B.J., Dwarakanath V., Chikka B.K. (2015). Streptozotocin—A diabetogenic agent in animal models. Int. J. Pharm. Pharm. Res..

[34] Qiu T.A., Nguyen T.H.T., Hudson-Smith N.V., Clement P.L., Forester D.-C., Frew H., Hang M.N., Murphy C.J., Hamers R.J., Feng Z.V. (2017). Growth-Based Bacterial Viability Assay for Interference-Free and High-Throughput Toxicity Screening of Nanomaterials. Anal. Chem..

[35] Jacobson G.R., Poy F., Lengeler J.W. (1990). Inhibition of Streptococcus mutans by the antibiotic streptozotocin: Mechanisms of uptake and the selection of carbohydrate-negative mutants. Infect. Immun..

[36] Liu H.M., Sato Y., Tsuda Y. (1993). Chemistry of Oxo-Sugars.2. Regioselective and Stereoselective Synthesis of Methyl D-Hexopyranosiduloses and Identification of their Forms Existing in Solutions. Chem. Pharm. Bull..

[37] CLSI (2012). Methods for Dilution Antimicrobial Susceptibility Tests for Bacteria that Grow Aerobically.

