# Supplementary material for: Selective Modification of Streptozotocin at the C3 Position to Improve Its Bioactivity as Antibiotic and Reduce Its Cytotoxicity towards Insulin-Producing β Cells
Source: Antibiotics (Basel). 2020 Apr 15;9(4):182. doi: 10.3390/antibiotics9040182 (PMC7235802; doi:10.3390/antibiotics9040182)
Supplement: Supplementary file 1 [file antibiotics-09-00182-s001.pdf]

antibiotics

MDPI

Selective Modification of Streptozotocin at the C3 Position to Improve Its Bioactivity as Antibiotic and Reduce Its Cytotoxicity towards Insulin-Producing β Cells

Ji Zhang,[a,†] Liubov Yakovlieva,[a,†] Bart J. de Haan,[b] Paul de Vos,[b] Adriaan J. Minnaard,[a] Martin D. Witte,[a] Marthe T.C. Walvoort[a,*]

Correspondence: Dr. Marthe T.C. Walvoort, m.t.c.walvoort@rug.nl

Supplementary Information

Supplementary Figures……………………………………………………………….………S-2

General Experimental Procedures……………………………………………………………..S-4

Chemical Synthesis of Compounds 2–6…………………………………………………...S-4

MIC Assay………………………………………………………………………………….……S-7

Viability Assay…………………………………………………………………………..…S-8

Cytotoxicity Assay……………………………………………………………………...…S-9

References……………………………………………………………………………..S-9

NMR, IR and HRMS Spectra……………………………………………………..…………..…S-10

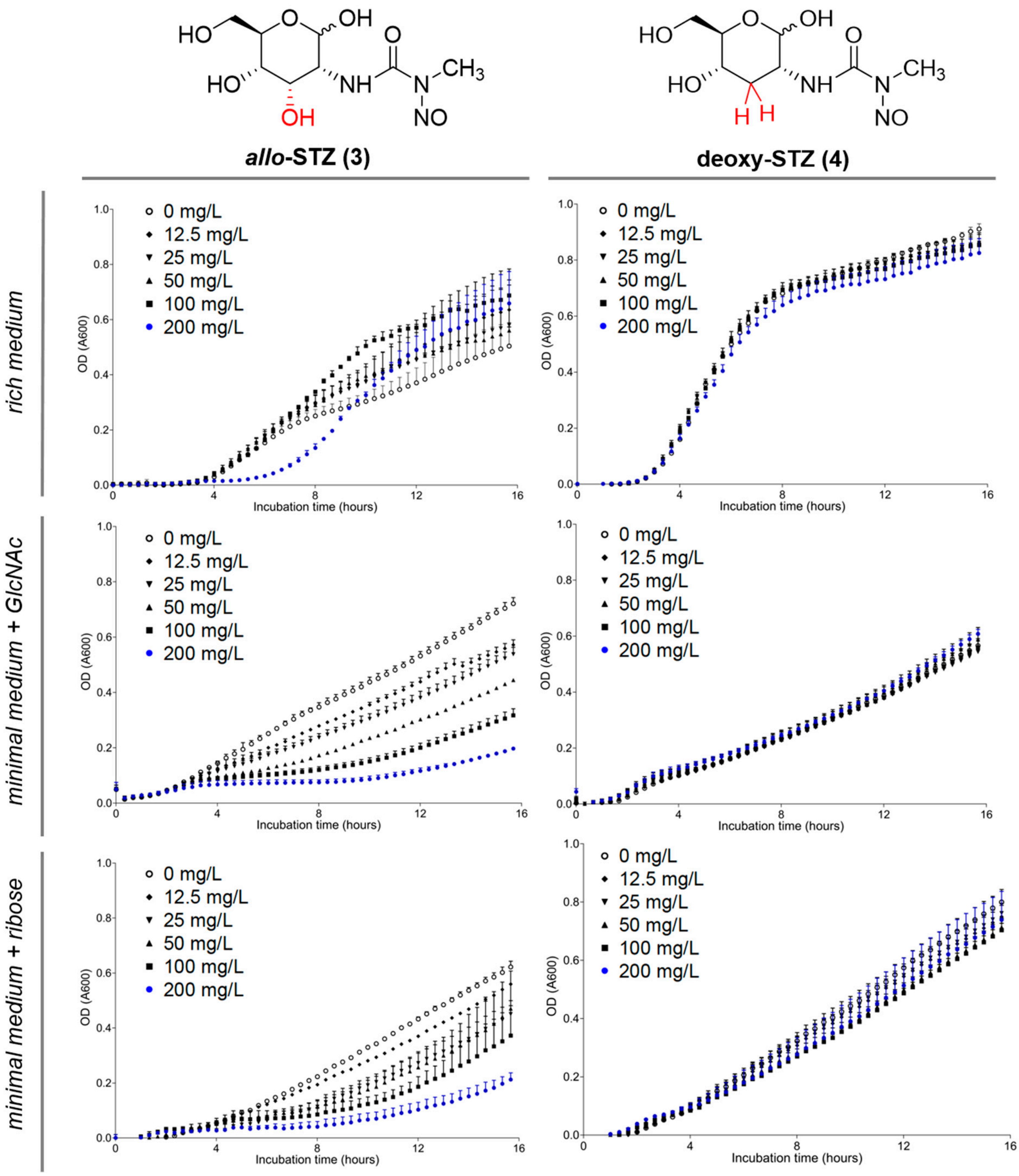

Figure S1. Growth curves of *E. coli* TOP10 to determine the MIC value for *allo*-STZ **3** (*top left*) and deoxy-STZ **4** (*top right*) in rich medium, *allo*-STZ **3** (*middle left*) and deoxy-STZ **4** (*middle right*) in minimal medium + GlcNAc, and *allo*-STZ **3** (*bottom left*) and deoxy-STZ **4** (*bottom right*) in minimal medium + ribose. Experiments are performed in triplicate.

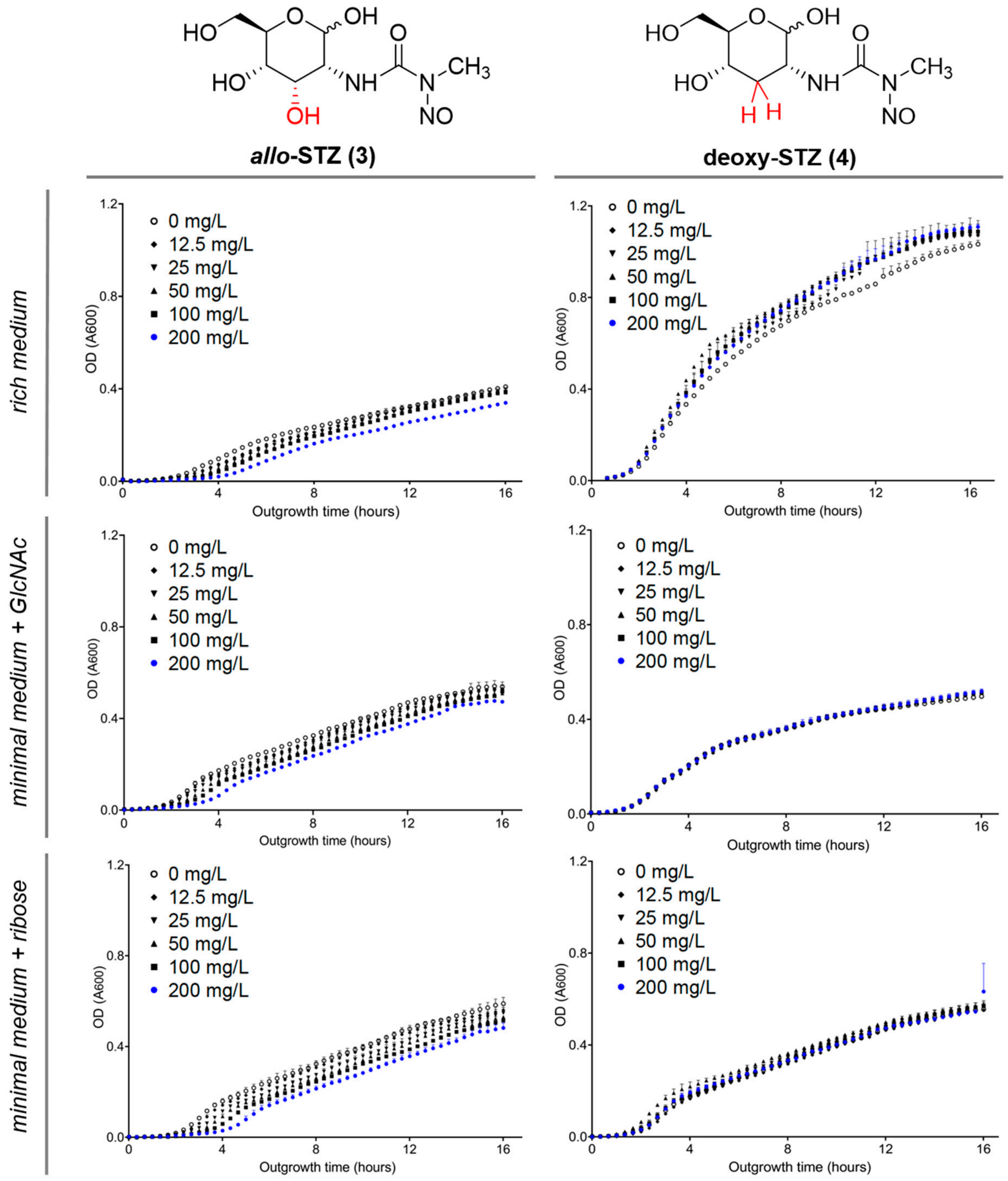

Figure S2. Growth-based viability curves of *E. coli* TOP10 for *allo*-STZ **3** (*top left*) and deoxy-STZ **4** (*top right*) in rich medium, *allo*-STZ **3** (*middle left*) and deoxy-STZ **4** (*middle right*) in minimal medium + GlcNAc, and *allo*-STZ **3** (*bottom left*) and deoxy-STZ **4** (*bottom right*) in minimal medium + ribose. Experiments are performed in duplicate.

General Experimental Procedures

All solvents used for reaction, extraction, filtration, and chromatography were of commercial grade and used without further purification. Flash chromatography was performed on a Reveleris® X2 Flash Chromatography, using Grace® Reveleris Silica flash cartridges (4 grams, 12 grams, 15 grams, 24 grams, 40 grams, 80 grams and 120 grams) and Scorpius Diol (OH) 48 grams. ^{1}H-, ^{13}C-, APT-, HSQC-, and COSY-NMR were recorded on a Varian AMX400 spectrometer (400, 100 MHz, respectively) using DMSO-d_6, D_2O or methanol-d_4 as solvent. Chemical shift values are reported in ppm with the solvent resonance as the internal standard (DMSO-d_6: δ 2.50 for ^{1}H, δ 39.52 for ^{13}C, CD_3OD: δ 3.31 for ^{1}H, δ 49.15 for ^{13}C; D_2O: δ 4.80 for ^{1}H). Data are reported as follows: chemical shifts (δ), multiplicity (s = singlet, d = doublet, dd = double doublet, ddd = double double doublet, t = triplet, appt = apparent triplet, q =quartet, m = multiplet), coupling constants J (Hz), and integration. High Resolution Mass measurements were performed using a ThermoScientific LTQ OribitrapXL spectrometer. Streptozotocin was obtained from Sigma-Aldrich (and prepared as well). *E. coli* TOP10 was obtained from the group of Biotransformations and Biocatalysis (GBB Institute, University of Groningen). Media, salts and additives were purchased from Sigma Aldrich, unless otherwise specified.

Chemical Synthesis of STZ Analogs 2–4, and Compounds 5 and 6

Keto-streptozotocin (2)

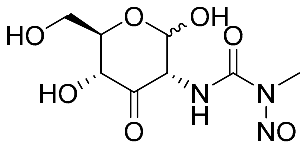

Streptozotocin (162 mg, 0.611 mmol, α/β >9/1) and benzoquinone (99 mg, 0.92 mmol) were dissolved in DMSO (2 mL). The catalyst $[(neocuproine)PdOAc]_2OTf_2$ (15.7 mg, 2.5 mol%) was added and the mixture was stirred at r.t. for 1 h. Upon completion of the reaction (according to TLC), water (20 mL) was added and the mixture was lyophilized to afford the crude product. Subsequent purification by flash chromatography on a 12 g silica cartridge with pentane/EtOAc, increasing the ratio of EtOAc from 0% to 100% in 22 min, the fraction eluting at 100% EtOAc afforded keto-streptozotocin **2** as a white solid (88 mg, 58%). HRMS (ESI) m/z calcd for keto-streptozotocin **2** $C_8H_{14}N_3O_7$ ($[M+H]^+$): 264.083 and the oxacarbium ion $C_8H_{12}N_3O_6$ ($[M-OH]^+$): 246.072; found: 264.083 and 246.072. ^{1}H NMR (400 MHz, Methanol-d_4) δ 5.71 (d, *J* = 4.1 Hz, 1H), 4.94 (d, *J* = 4.1 Hz, 1H), 4.40 (d, *J* = 9.7 Hz, 1H), 4.00 (dt, *J* = 9.7, 3.3 Hz, 1H), 3.90–3.85 (m, 2H), 3.16 (s, 3H); ^{13}C NMR (101 MHz, Methanol-d_4) δ 204.5, 95.3, 76.8, 73.9, 62.6, 61.9, 26.9; IR (N-N=O): 1435 cm^{-1}

Allo-streptozotocin (3)

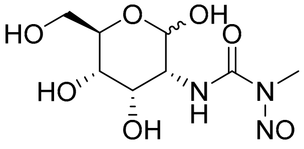

Allosamine hydrochloride (393 mg, 1.82 mmol) and 4-nitrophenyl *N*-nitroso-*N*-methylcarbamate (451 mg, 2 mmol) were dissolved in DMF (12 mL) at r.t., then the reaction mixture was cooled to 0 °C under N_2 atmosphere, followed by the addition of *N*,*N*-diisopropylethylamine (283 mg, 2.19 mmol). The reaction mixture was stirred at 0 °C for 2 h, the DMF was evaporated and the residue purified by flash chromatography on a 12 g silica cartridge with EtOAc/MeOH, increasing the ratio of MeOH from 0% to 10% in 22 min, the product eluted at 3% MeOH to afford a yellow solid (200 mg, 41%). NMR showed the major form is β-pyranose, in approximately 48%. $[\alpha]_D$=10.7° (*c* = 0.0101, CH_3OH); HRMS (ESI) m/z calcd for *allo*-streptozotocin **3** $C_8H_{16}N_3O_7$ ($[M+H]^+$): 266.098 and the oxacarbenium ion $C_8H_{14}N_3O_6$ ($[M-OH]^+$): 248.088; found: 266.097 and 248.087; ^{1}H NMR (400 MHz, Methanol-d_4) δ 4.96 (d, *J* = 8.3 Hz, 1H), 4.16 – 4.13 (m, 1H), 3.91 (dd, *J* = 8.3, 2.9 Hz, 1H), 3.86 (dd, *J* = 11.7, 2.5 Hz, 1H), 3.81–3.75 (m, 1H), 3.69 (dd, *J* = 11.6, 5.7 Hz, 1H), 3.60 (dd, *J* = 9.6, 3.1 Hz, 1H), 3.16 (s, 3H); ^{13}C NMR (101 MHz, Methanol-d_4) δ 153.5, 93.2, 74.4, 70.1, 67.4, 61.8, 55.6, 25.4; IR (N-N=O): 1436 cm^{-1}

Deoxy-streptozotocin (4)

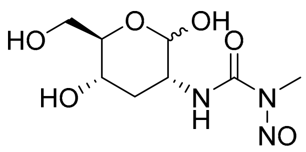

Lividosamine hydrochloride (154 mg, 0.771 mmol) and 4-nitrophenyl *N*-nitroso-*N*-methylcarbamate (191 mg, 0.848 mmol) were dissolved in DMF (5 mL) at r.t., then the reaction mixture was cooled to 0 °C under N_2 atmosphere, followed by the addition of *N*,*N*-diisopropylethylamine (161 µL, 0.925 mmol), The reaction mixture was stirred at 0 °C for 2 h, the DMF was evaporated and the residue purified by flash chromatography on a 15 g silica cartridge with 100% EtOAc to afford the product (135 mg, 70%) as colorless oil. NMR showed the major form is α-pyranose, in approximately 60%. $[\alpha]_D$ = 34.7° (*c* = 0.0117, CH_3OH); HRMS (ESI) m/z calcd for deoxy-streptozotocin **4** $C_8H_{15}N_3O_6$ ($[M+H]^+$): 250.103 and the oxacarbenium ion $C_8H_{14}N_3O_5$ ($[M-OH]^+$): 232.093; found: 250.103 and 232.093; ^1H NMR (400 MHz, Methanol-d_4) δ 5.17 (d, *J* = 3.4 Hz, 1H), 4.12 (dt, *J* = 12.7, 4.1 Hz, 1H), 3.83 – 3.78 (m, 1H), 3.73–3.70 (m, 1H), 3.69 (q, *J* = 6.0 Hz, 1H), 3.63-3.59 (m, 1H), 2.15 (dt, *J* = 11.6, 4.7 Hz, 1H), 1.90 (q, *J* = 11.8 Hz, 1H); ^{13}C NMR (101 MHz, Methanol-d_4) δ 154.7, 91.2, 74.1, 66.3, 62.9, 51.0, 34.1, 26.9; IR (N-N=O): 1478 cm^{-1}

N-methyl 4-nitrophenyl carbamate (5)

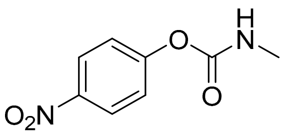

Methyl amine (12 mmol, 6 mL of a 2 M solution in THF) was added to a solution of 4-nitrophenyl chloroformate (2.0 g, 9.9 mmol) in THF (100 mL) at 0 °C. The reaction mixture was allowed to warm to r.t. and stirred overnight. The mixture was then concentrated and purified by flash chromatography on a silica cartridge with PE/EtOAc to obtain the product (1.07 g, 55%) as a white solid, m.p.: 144–146 °C (lit.[1] 145–146 °C); ^{1}H NMR (400 MHz, Chloroform-*d*) δ 8.24 (d, *J* = 9.0 Hz, 2H), 7.31 (d, *J* = 9.0 Hz, 2H), 5.07 (s, 1H), 2.93 (d, *J* = 4.7 Hz, 3H); ^{13}C NMR (101 MHz, Chloroform-*d*) δ 156.0, 153.7, 144.7, 125.1, 121.9, 27.8.

4. -nitrophenyl *N*-nitroso-*N*-methylcarbamate (6)

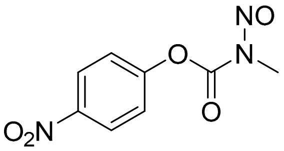

To a solution of *N*-methyl 4-nitrophenyl carbamate (1 g, 5.1 mmol) in 20 mL DCM in a 100 mL flask, was added a solution of $NaNO_2$ (2.5 g, 36.2 mmol) in 20 mL of water. The mixture was chilled to 0 °C, and 5.2 mL conc. HCl aq was added dropwise and slowly (one drop per 3 seconds), upon which the color of the solution changed from yellow to green. The reaction was monitored by TLC, and after 3 h, a very small amount of starting material remained. Nevertheless, the DCM layer was separated, washed with water, dried with $MgSO_4$, and concentrated. Purification by flash chromatography on a silica cartridge with pentane/DCM yielded a yellow solid (852 mg, 74%); HRMS (ESI) m/z calcd for $C_8H_8N_3O_5$ ($[M+H]^+$): 226.046 and $C_8H_7N_2O_4$ ($[M-NO]^+$): 195.040; found: 225.979 and 195.039; ^{1}H NMR (400 MHz, Chloroform-*d*) δ 8.36 (d, *J* = 9.2 Hz, 2H), 7.53 (d, *J* = 9.1 Hz, 2H), 3.26 (s, 3H); ^{13}C NMR (101 MHz, Chloroform-*d*) δ 154.8, 151.9, 145.9, 125.5, 122.3, 28.2; The NMR data are in accordance to those published [2]. IR (N-N=O): 1460 cm^{-1}

Minimal Inhibitory Concentration (MIC) Assays

MIC determination in rich medium. MIC determination was carried out in 96-well plates following the CLSI guidelines [3]. The pre-culture of *E. coli* TOP10 (K12 origin) was prepared from the glycerol stock in LB medium and incubated overnight at 37 °C and shaking. The next day this pre-culture was used to inoculate fresh LB medium and *E. coli* cells were grown to OD_{600} of 0.08–0.1 (turbidity of the 0.5 McFarland standard). The culture was subsequently diluted with LB medium to reach the density of approximately 5×10^6 CFU/mL and then 10 μL of the resulting suspension were transferred to the exposure wells (total volume 100 μL), yielding the final culture density of 5×10^5 CFU/mL. The serial dilutions of the compounds were prepared in 10× concentrated form, and 10 μL of each respective series were transferred to the exposure well (100 μL final volume) resulting in 1× concentrated series. Within 15min of adding the inoculum the 96-well plate was incubated at 37 °C for 16–22h in an plate-reader (Synergy™ 2 Multi-Mode Microplate Reader, BioTek Instruments) under ambient air with OD_{600} measurements every 20 min preceded by 30 s of shaking.

MIC determination in minimal medium with pre-sensitizing. The pre-culture of *E. coli* TOP10 (K12 origin) was prepared from glycerol stock in minimal medium (50mM Na_2HPO_4, 25mM KH_2PO_4, 10mM NaCl, 5mM $MgSO_4$, 0.2mM $CaCl_2$, 50uM $FeCl_3$, 0.1% NH_4Cl, 1% casein hydrolysate) in the presence of 4% (w/v) of the pre-sensitizer (*N*-acetylglucosamine or ribose) and incubated overnight at 37 °C with shaking. The next day cells were pelleted by centrifugation and washed with PBS (phosphate buffer saline) three times to remove the traces of presensitizer. After the final washing step pellet was resuspended in 5 milliliters of PBS and used to inoculate minimal medium containing 1% glycerol. The resulting culture was allowed to grow until OD_{600} of 0.08–0.1 (turbidity of the 0.5 McFarland standard). The culture was subsequently diluted with minimal medium to reach the density of approximately 5×10^6 CFU/mL and then 10 μL of the resulting suspension were transferred to the exposure wells (total volume 100 μL), yielding the final culture density of 5×10^5 CFU/mL. The serial dilutions of the compounds were prepared in 10x concentrated form and 10 μL of each respective series were transferred to the exposure well (100 μL final volume) resulting in 1× concentrated series. Within 15min of adding the inoculum the 96-well plate was incubated at 37 °C for 16–22h in an ambient air incubator with OD_{600} measurements every 20 min preceded by 30 s of shaking.

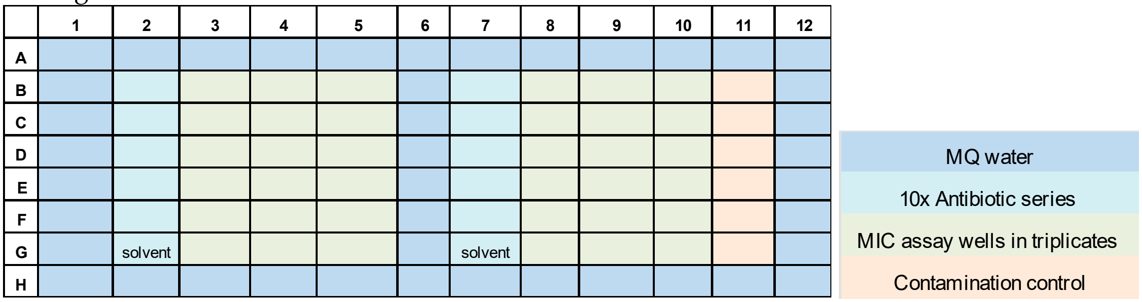

Viability Assays

Growth-based viability assay. In order to determine the inhibitory effect of streptozotocin and its derivatives, the growth-based viability assay developed by C.L. Haynes et al. [4] was used. The underlying principle of the assay is to estimate the viability of the cells after exposure to an inhibitory compound by the delay in the subsequent outgrowth in fresh medium. This effect stems from the fact that the fewer the remaining cells, the longer it takes to reach a certain density threshold, indicating the bactericidal activity of the compound used. In the assay, bacterial cells were first exposed to the streptozotocin derivatives (either in rich or minimal medium) at room temperature and constant shaking to ensure good mixing. Afterwards, a small fraction of the exposure mixture was transferred into the fresh medium (yielding a 40× dilution and therefore alleviating the effect of the antibiotic) and the cells were incubated in a Biotek plate reader for 16 hours at 37 °C with optical density measurements at 600 nm taking place every 20 min preceded by 30 s of shaking.

Plate layout. As recommended in the paper of Haynes[4] the original layout with water evaporation control was used in this work. For all experiments the following plate sections were included: calibration series with outgrowth in duplicates, 10× dilution series of the compound, exposure wells and after-exposure outgrowth wells in duplicates.

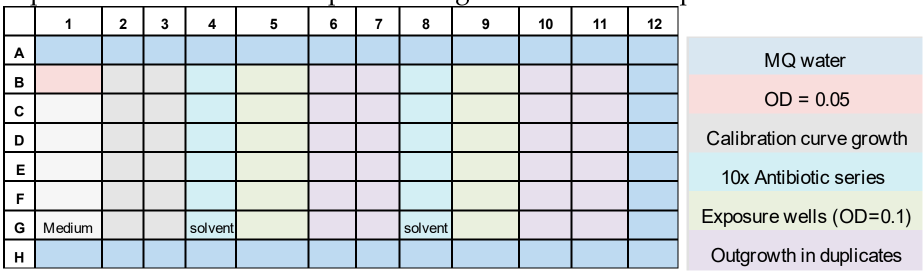

Viability assay in rich medium. Bacterial culture of *E. coli* TOP10 was grown in LB medium (37 °C, shaking) until cells entered the exponential growth phase (OD_{600} 0.3-0.5) and was diluted to working density of 0.05 (calibration curve series) and 0.1 (exposure). For exposure, 20 µL of the 10× compound preparation in an appropriate solvent was transferred to 180 µL of the bacterial culture, yielding 1× concentration of the compound for exposure. The plate was then incubated in the plate reader at room temperature and constant shaking for 2 hours. Afterwards, 5 µL of the exposure mixture were added to 195 µL of fresh LB to dilute the antibiotic and allow remaining bacterial cells to outgrow. The same outgrowth procedure was performed for the calibration series. As a last step, the plate was incubated for 16 hours in the plate reader at 37 °C with OD_{600} measurements every 20 min preceded by 30 s of shaking.

Viability assay in minimal medium on pre-sensitized *E. coli* cells. Bacterial culture of *E. coli* TOP10 was pre-grown overnight in minimal medium (50mM Na_2HPO_4, 25mM KH_2PO_4, 10mM NaCl, 5mM $MgSO_4$, 0.2mM $CaCl_2$, 50uM $FeCl_3$, 0.1% NH_4Cl, 1% casein hydrolysate) containing 0.4% *N*-acetylglucosamine or ribose at 37 °C with shaking. Next day, cells were harvested and washed three times with Dulbecco’s phosphate saline buffer (DPBS) and resuspended in the DPBS to the original volume of the overnight culture. The resulting bacterial suspension was then used to inoculate minimal medium containing 1% glycerol which was further incubated at 37 °C and shaking. The bacterial culture was allowed to reach exponential phase densities of 0.3–0.5 and then was diluted to working densities of 0.05 (calibration curve) and 0.1 (exposure). Exposure and outgrowth procedures were performed in the same way as described in the section “Viability assay in rich medium”.

Calculation of viability. The procedure to calculate viability was taken from the original article by Haynes et al [4]. In short, the viability of the bacterial culture after exposure to the STZ analogues is defined as the percentage of cells remaining. This percentage is calculated from a calibration curve of defined optical densities (OD at 600 nm, related to percentage of cells), and their associated delays in time for outgrowth above a threshold OD (defined as Ct).

To construct the calibration curve, a serial dilution was performed to obtain 100%, 50%, 25%, 12.5%, 6.25% of total viable cells. Outgrowth was performed as described above and monitored over time with 20 minute intervals (defined as cycles). A plot was generated of the cycle number

(equivalent to 20 minutes) versus the log_{10} of the corrected OD values. To determine the Ct number at which the threshold cell density is crossed for each dilution series, the linear part of the curves and the equations describing them was used. The log_{10} value of 0.02 (cell density threshold) was plugged into the equations to obtain Ct numbers for the range of viability (v = 1 , 0.5, 0.25, 0.125, 0.0625). Next, the viability values were converted into dilution fold values ($\mathrm{d} = -\log_2 \mathrm{v}$) and plotted against Ct values calculated earlier. Obtained linear graph and equation that describes it were later used to determine d-values (and subsequently viability from the formula $v = 2^{-d}$) by plugging in experimental Ct.

Cytotoxicity Assay

Cell Culture

The MIN6 cell line was purchased from American Type Culture Collection (ATCC, Manassas, USA). MIN6 cells (passages 30-45) were cultured in DMEM High glucose medium (Lonza, Basal, Switzerland), containing 15% fetal bovine serum (FBS, Lonza), 50 µmol/L β-mercaptoethanol, 2 mmol/L L-glutamine, 50 U/mL penicillin, and 50 µg/mL streptomycin (all from Sigma-Aldrich, St. Louis, USA). Cells were cultured at 37 °C in a humidified atmosphere containing 95% air and 5% CO_2.

Cell Viability

The effect of streptozotocin (STZ) and the derivatives on β-cell viability was determined by the cell proliferation reagent WST-1 (Roche, Indianapolis, USA). Briefly, MIN6 cells (1×10^5 cells/well) were seeded in 96-well plates. Cells were cultured overnight and the following day incubated with or without STZ or analog (Sigma-Aldrich) at 5 mM for 48 and 72 hours followed by WST-1 assay. After 30 min incubation with WST-1 (10 µL/well) at 37 °C, the absorbance was measured at 450 nm using a Bio-Rad Benchmark Plus microplate spectrophotometer reader (Bio-Rad Laboratories B.V, Veenendaal, the Netherlands).

References

1. Al-Rawi, H.; Williams, A. Elimination-Addition Mechanisms of Acyl Group Transfer - Hydrolysis and Synthesis of Carbamates. *J. Am. Chem. Soc.* **1977**, *99*, 2671-2678.
2. Mori, R.; Kato, A.; Komenoi, K.; Kurasaki, H.; Iijima, T.; Kawagoshi, M.; Kiran, Y. B.; Takeda, S.; Sakai, N.; Konakahara, T. Synthesis and in vitro antitumor activity of novel 2-alkyl-5-methoxycarbonyl-11-methyl-6H-pyrido[4,3-b]carbazol-2-ium and 2-alkylellipticin-2-ium chloride derivatives. *Eur. J. Med. Chem.* **2014**, *82*, 16-35.
3. CLSI *Methods for Dilution Antimicrobial Susceptibility Tests for Bacteria That Grow Aerobically; Approved Standard—Ninth Edition;* Clinical and Laboratory Standards Institute: 2012; .
4. Qiu, T. A.; Thu Ha Thi Nguyen; Hudson-Smith, N. V.; Clement, P. L.; Forester, D.; Frew, H.; Hang, M. N.; Murphy, C. J.; Hamers, R. J.; Feng, Z. V.; Haynes, C. L. Growth-Based Bacterial Viability Assay for Interference -Free and High-Throughput Toxicity Screening of Nanomaterials. *Anal. Chem.* **2017**, *89*, 2057-2064.

Streptozotocin (1)

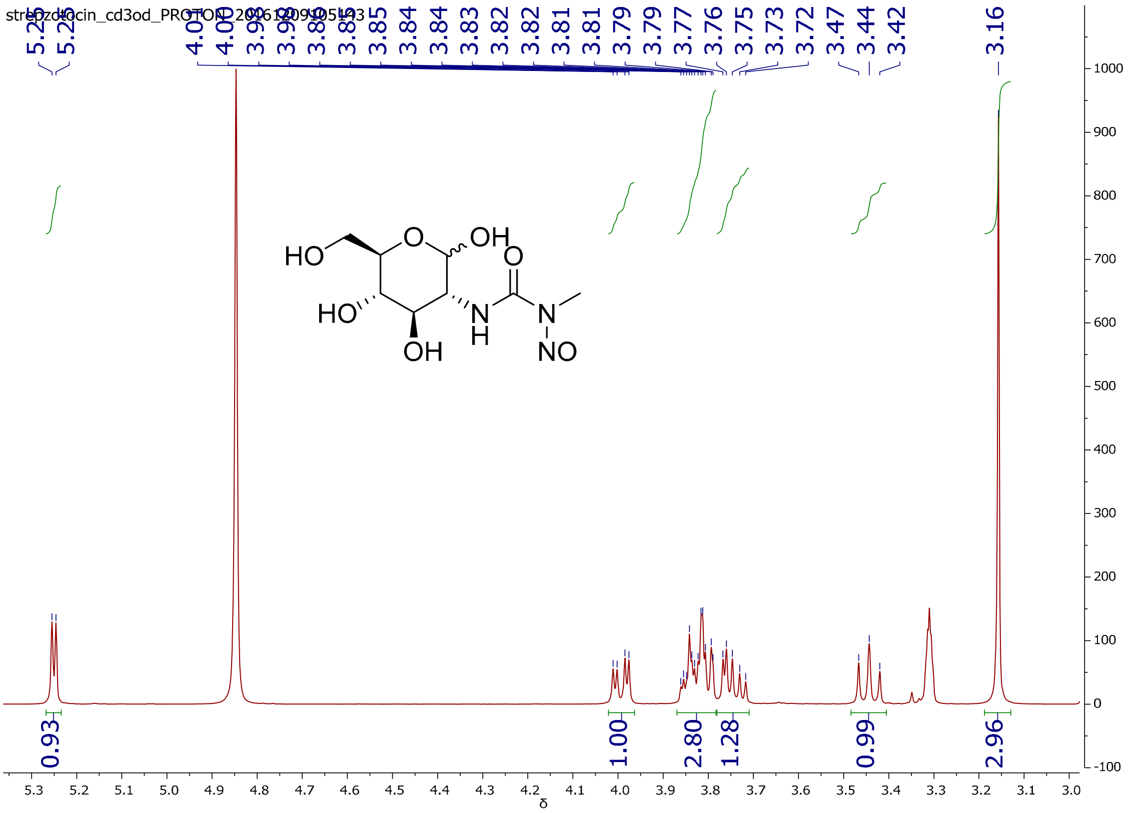

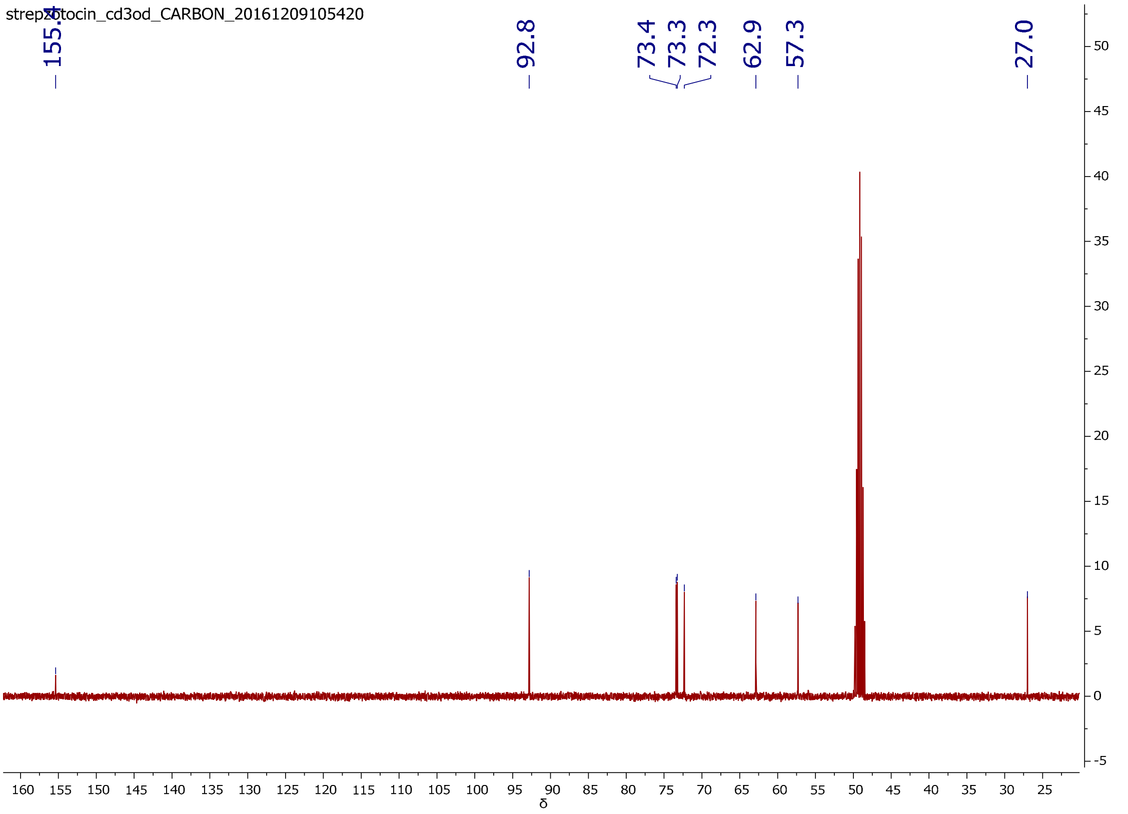

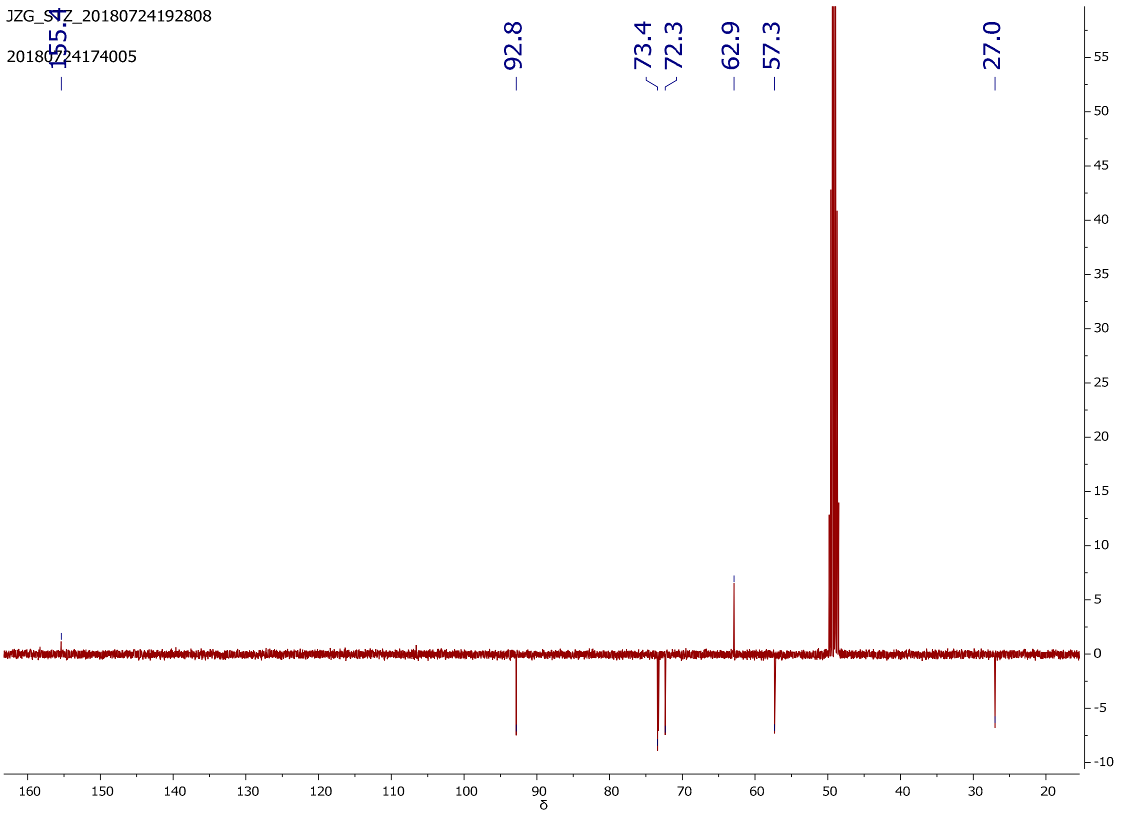

JZG_STZ_20180724192808
20180724174005
155.4
92.8
73.4
72.3
62.9
57.3
27.0
δ

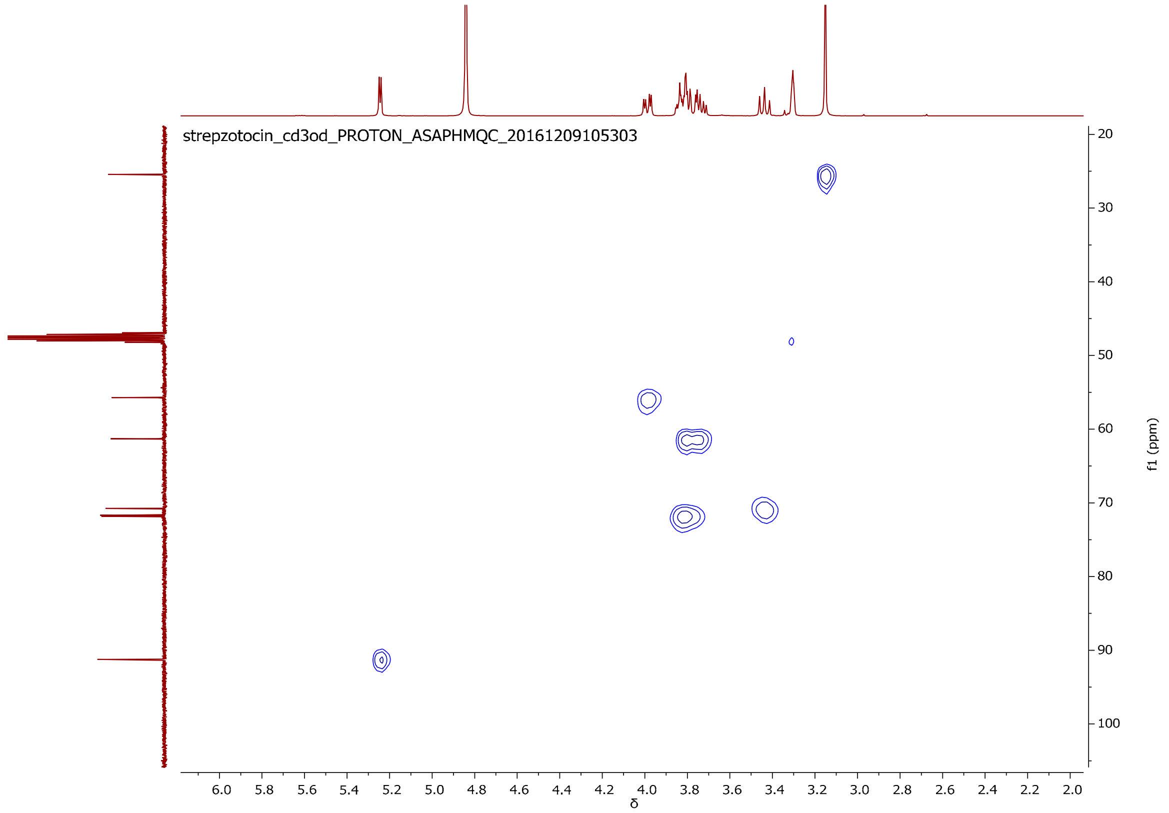

strepzotocin_cd3od_PROTON_ASAPHMQC_20161209105303
f1 (ppm)
δ

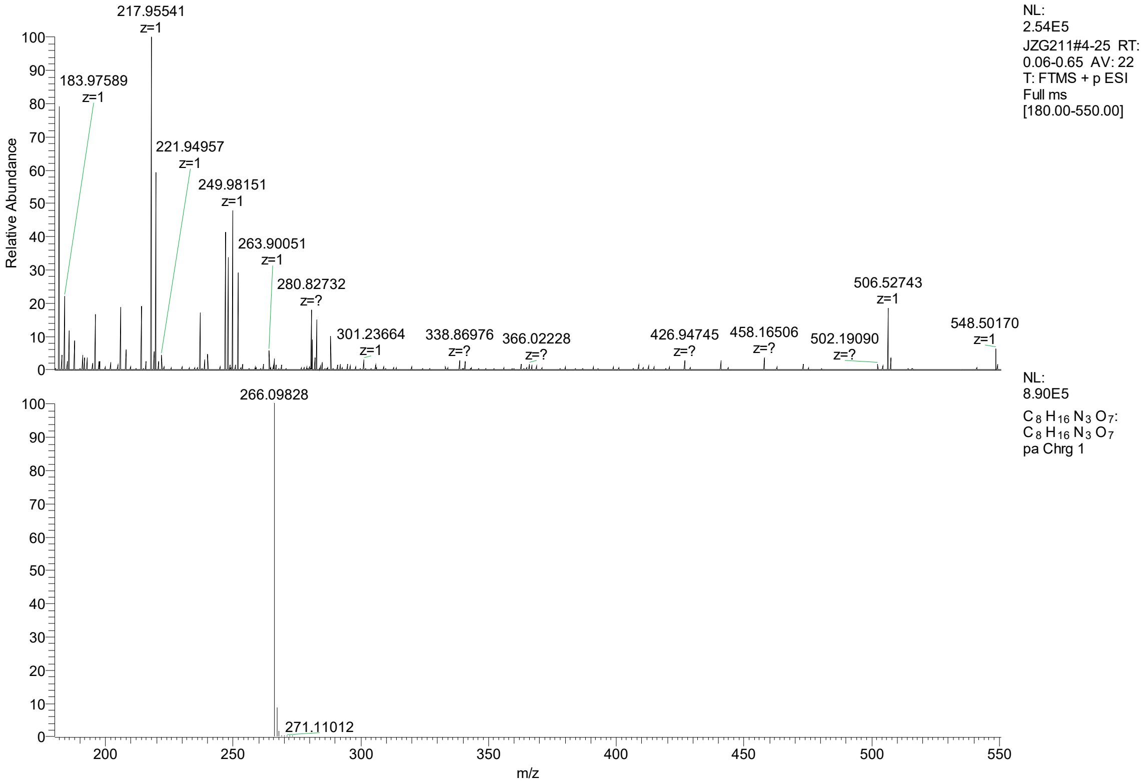

217.95541 z=1
183.97589 z=1
221.94957 z=1
249.98151 z=1
263.90051 z=1
280.82732 z=?
301.23664 z=1
338.86976 z=?
366.02228 z=?
426.94745 z=?
458.16506 z=?
502.19090 z=?
506.52743 z=1
548.50170 z=1
Relative Abundance
NL: 2.54E5
JZG211#4-25 RT: 0.06-0.65 AV: 22
T: FTMS + p ESI Full ms [180.00-550.00]
266.09828
271.11012
NL: 8.90E5
C8 H16 N3 O7: C8 H16 N3 O7 pa Chrg 1
m/z

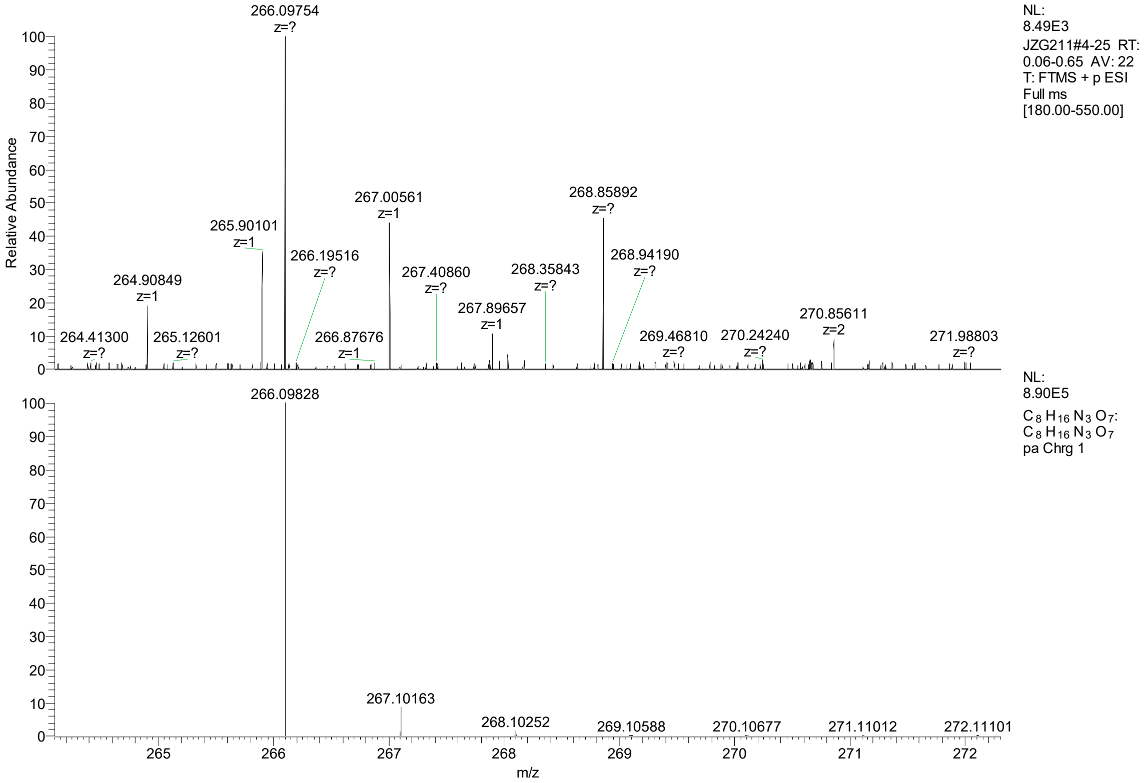

266.09754 z=?
265.90101 z=1
267.00561 z=1
268.85892 z=?
266.19516 z=?
267.40860 z=?
268.35843 z=?
268.94190 z=?
264.90849 z=1
267.89657 z=1
270.85611 z=2
264.41300 z=?
265.12601 z=?
266.87676 z=1
269.46810 z=?
270.24240 z=?
271.98803 z=?
Relative Abundance
NL: 8.49E3
JZG211#4-25 RT: 0.06-0.65 AV: 22
T: FTMS + p ESI Full ms [180.00-550.00]
266.09828
267.10163
268.10252
269.10588
270.10677
271.11012
272.11101
NL: 8.90E5
C8 H16 N3 O7: C8 H16 N3 O7 pa Chrg 1
m/z

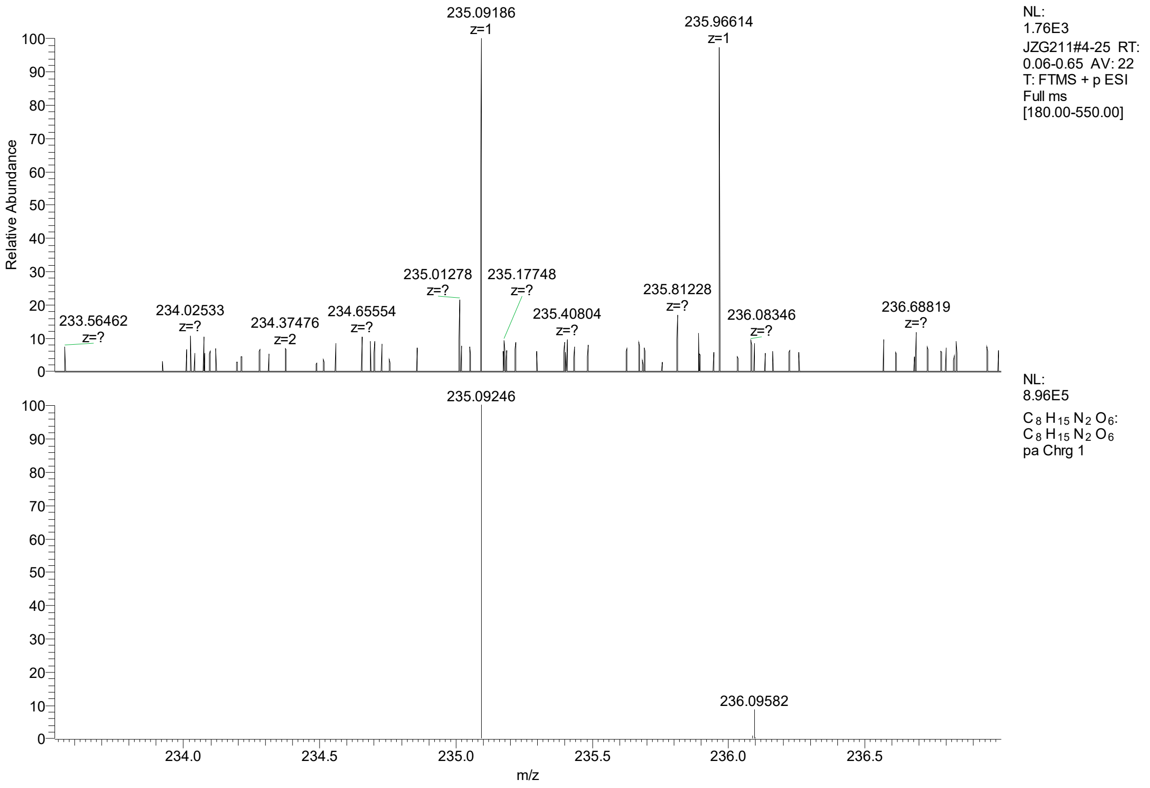
235.09186
z=1
235.96614
z=1
NL:
1.76E3
JZG211#4-25 RT:
0.06-0.65 AV: 22
T: FTMS + p ESI
Full ms
[180.00-550.00]
Relative Abundance
233.56462
z=?
234.02533
z=?
234.37476
z=2
234.65554
z=?
235.01278
z=?
235.17748
z=?
235.40804
z=?
235.81228
z=?
236.08346
z=?
236.68819
z=?
NL:
8.96E5
C 8 H 15 N 2 O 6:
C 8 H 15 N 2 O 6
pa Chrg 1
235.09246
236.09582
234.0
234.5
235.0
235.5
236.0
236.5
m/z

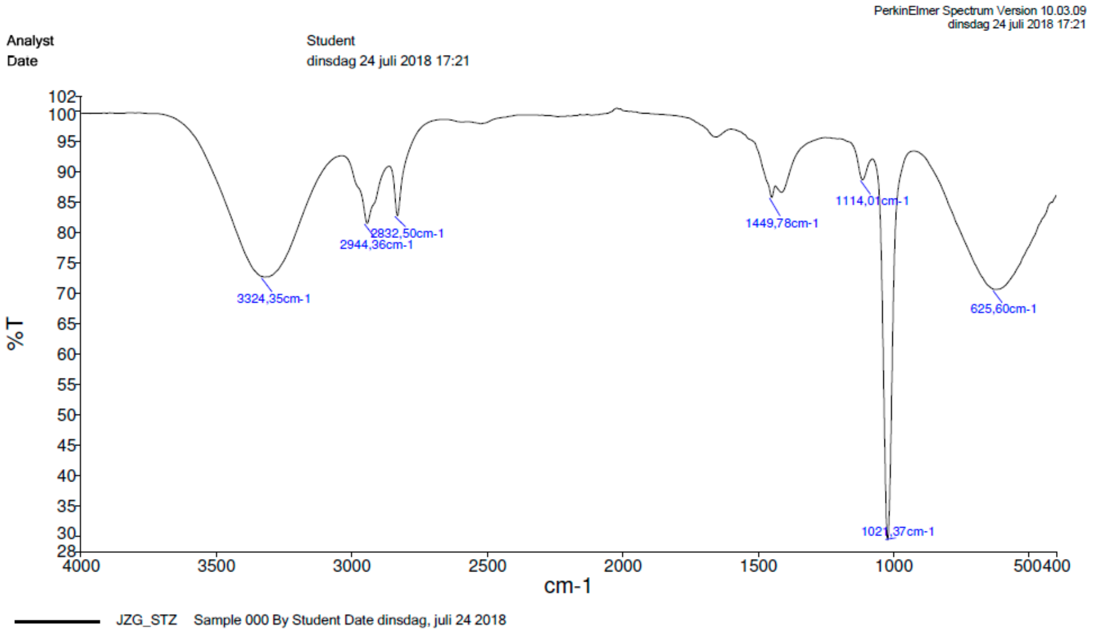
PerkinElmer Spectrum Version 10.03.09
dinsdag 24 juli 2018 17:21
Analyst
Date
Student
dinsdag 24 juli 2018 17:21
%T
3324,35cm-1
2944,36cm-1
2832,50cm-1
1449,78cm-1
1114,01cm-1
1021,37cm-1
625,60cm-1
4000
3500
3000
2500
2000
1500
1000
500400
cm-1
JZG_STZ
Sample 000 By Student Date dinsdag, juli 24 2018

Keto-streptozotocin (2)

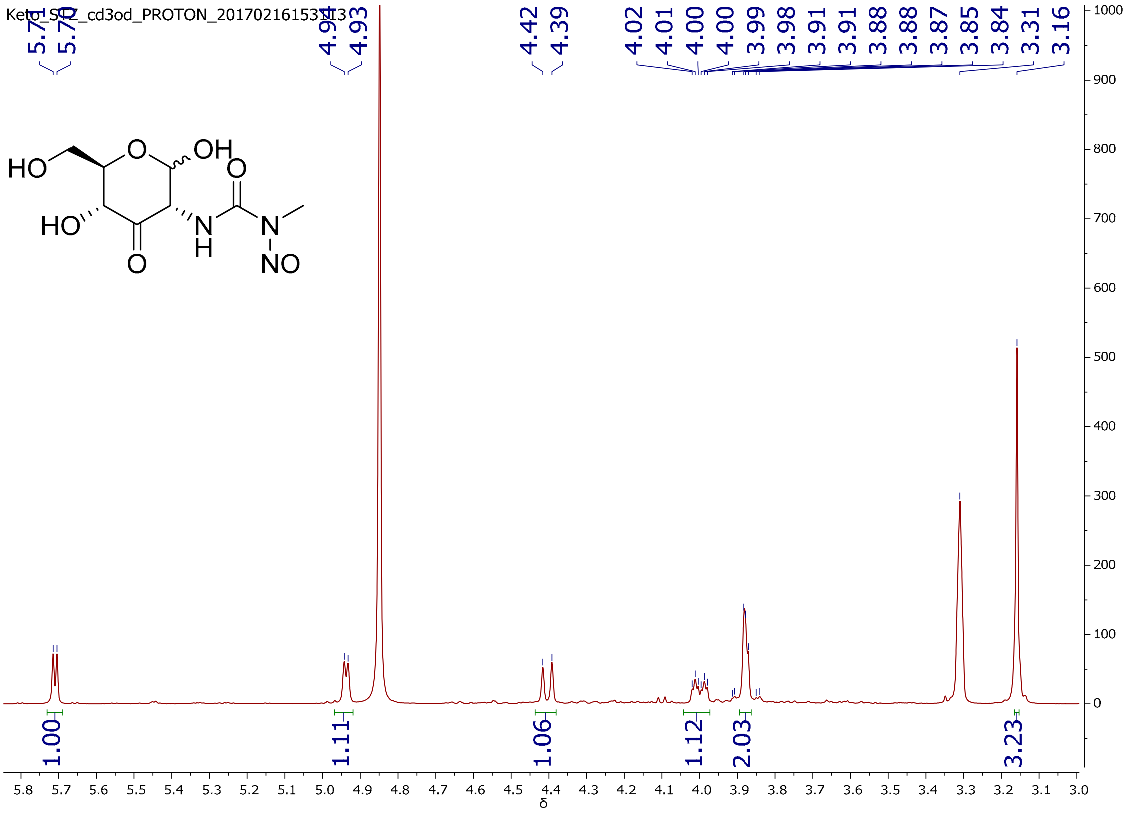

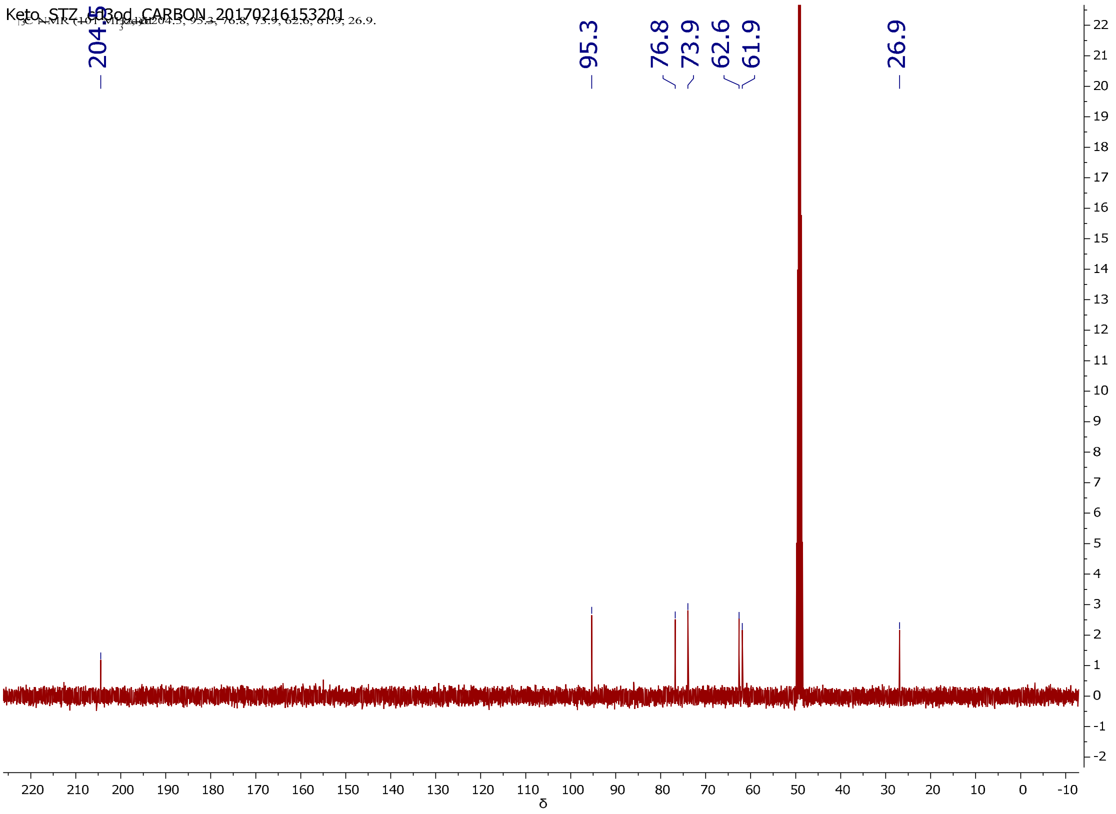

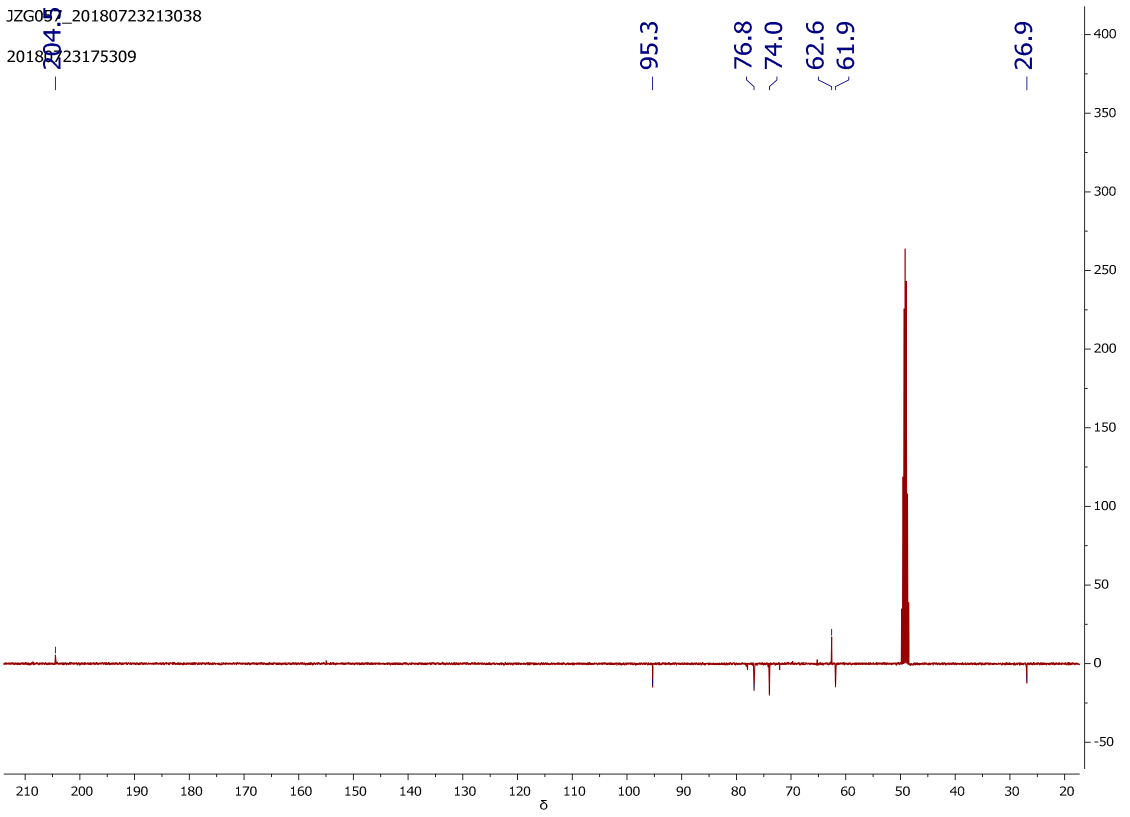

JZG057_20180723213038
20180723175309
204.5
95.3
76.8
74.0
62.6
61.9
26.9
δ

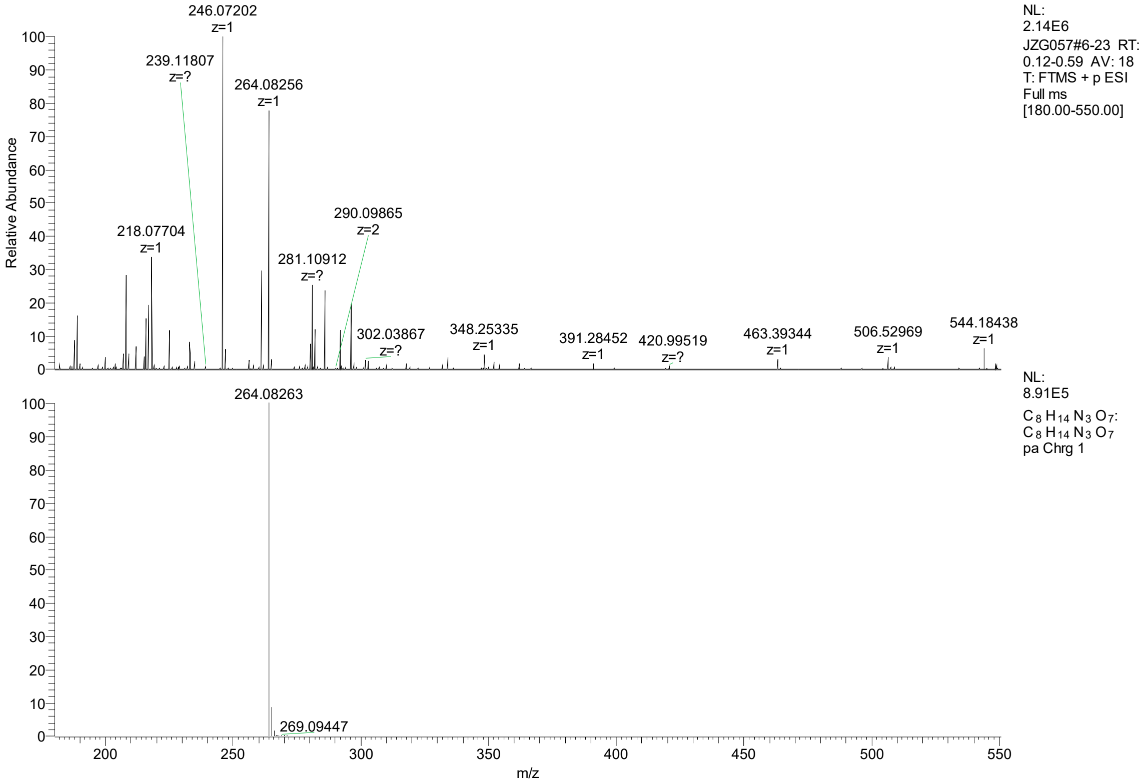

NL: 2.14E6
JZG057#6-23 RT: 0.12-0.59 AV: 18
T: FTMS + p ESI Full ms [180.00-550.00]
246.07202 z=1
239.11807 z=?
264.08256 z=1
218.07704 z=1
290.09865 z=2
281.10912 z=?
302.03867 z=?
348.25335 z=1
391.28452 z=1
420.99519 z=?
463.39344 z=1
506.52969 z=1
544.18438 z=1
NL: 8.91E5
C8 H14 N3 O7: C8 H14 N3 O7 pa Chrg 1
264.08263
269.09447
Relative Abundance
m/z

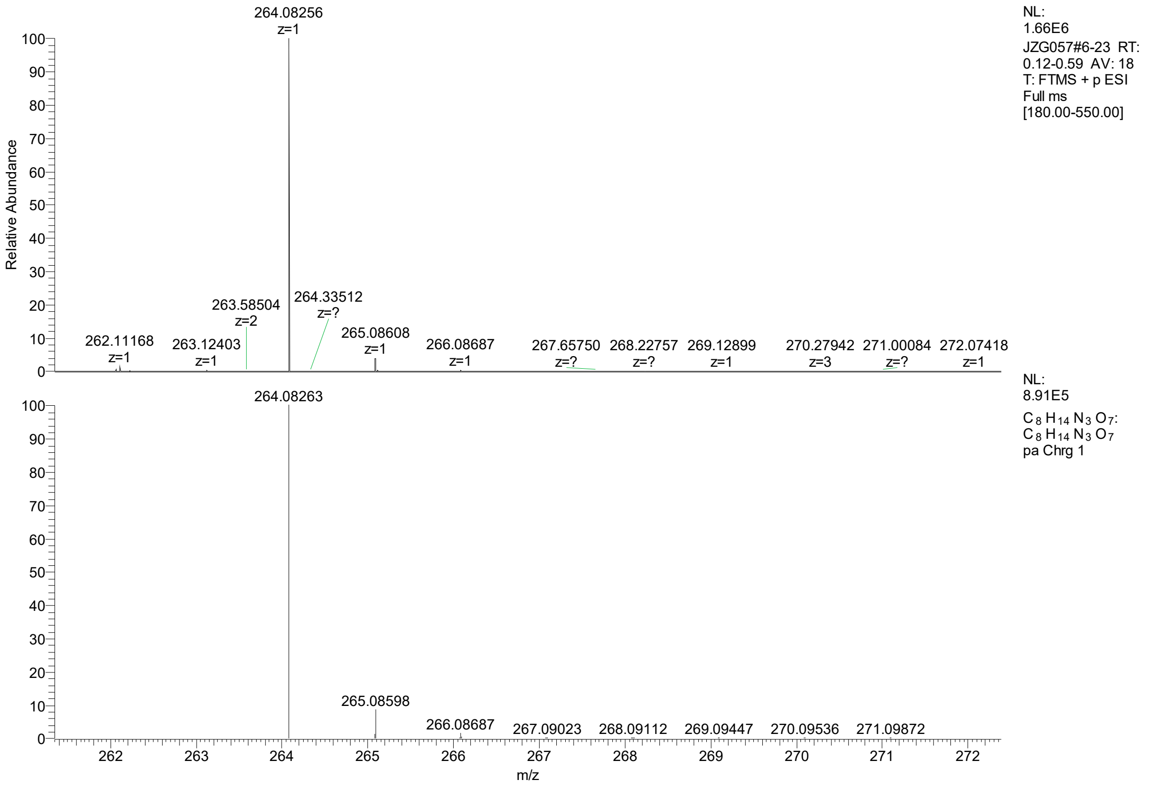
NL: 1.66E6
JZG057#6-23 RT: 0.12-0.59 AV: 18
T: FTMS + p ESI Full ms [180.00-550.00]
264.08256 z=1
263.58504 z=2
264.33512 z=?
262.11168 z=1
263.12403 z=1
265.08608 z=1
266.08687 z=1
267.65750 z=?
268.22757 z=?
269.12899 z=1
270.27942 z=3
271.00084 z=?
272.07418 z=1
Relative Abundance
NL: 8.91E5
C8 H14 N3 O7: C8 H14 N3 O7
pa Chrg 1
264.08263
265.08598
266.08687
267.09023
268.09112
269.09447
270.09536
271.09872
m/z

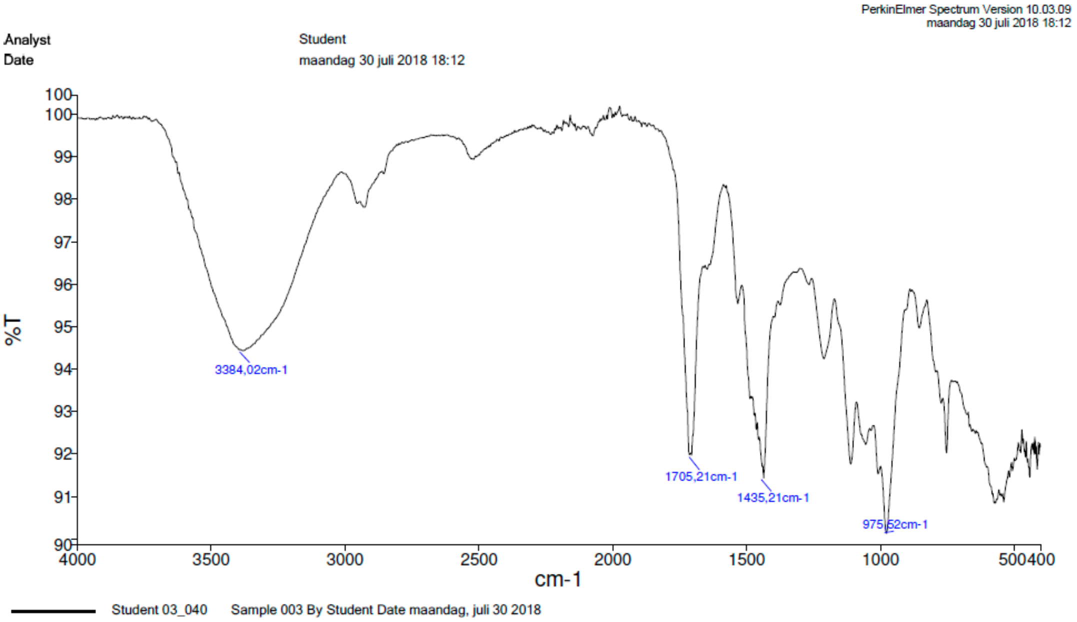
PerkinElmer Spectrum Version 10.03.09
maandag 30 juli 2018 18:12
Analyst Student
Date maandag 30 juli 2018 18:12
%T
3384,02cm-1
1705,21cm-1
1435,21cm-1
975,52cm-1
cm-1
Student 03_040 Sample 003 By Student Date maandag, juli 30 2018

***Allo*-streptozotocin (3)**

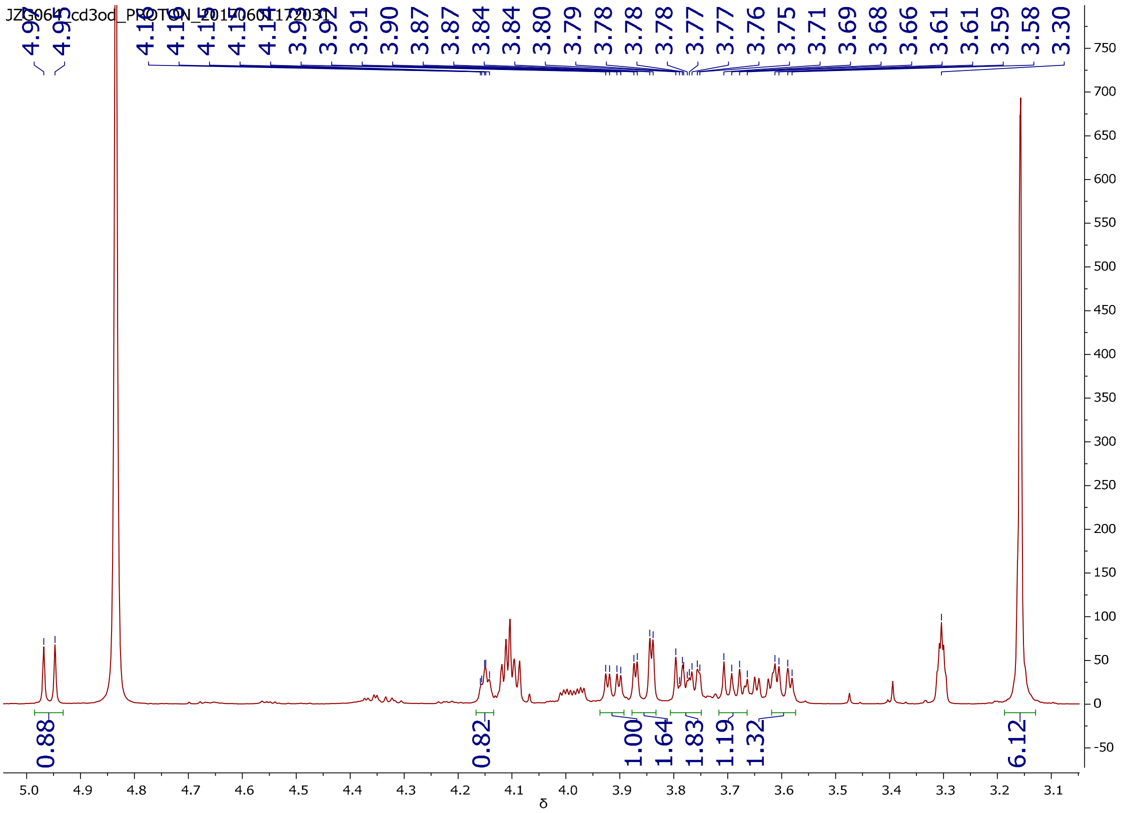

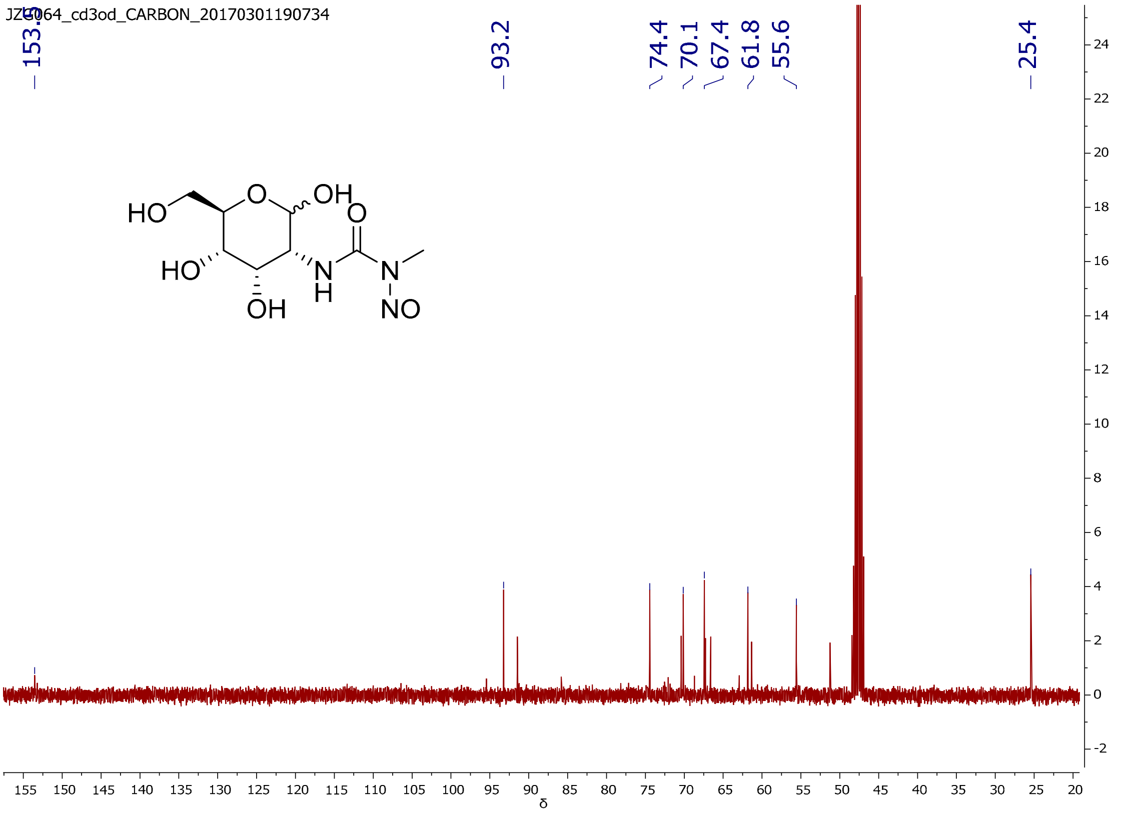

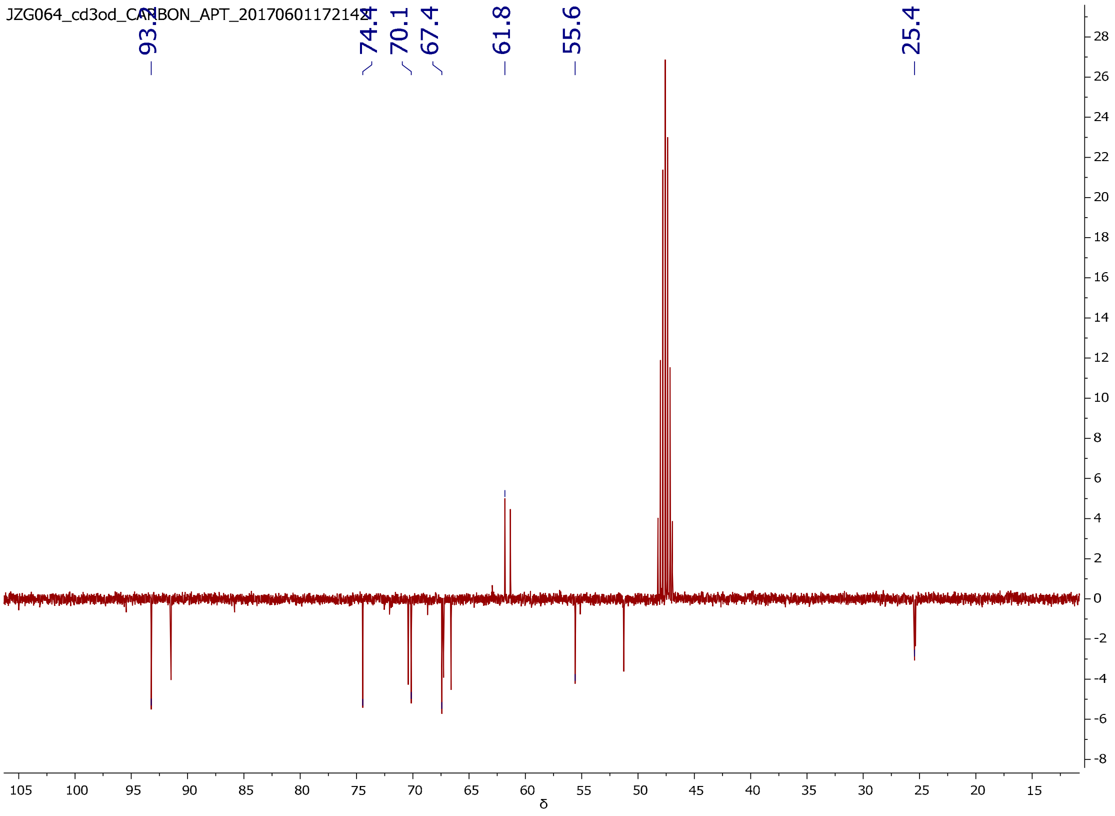
JZG064_cd3od_CARBON_APT_20170601172144
93.2
74.4
70.1
67.4
61.8
55.6
25.4
δ

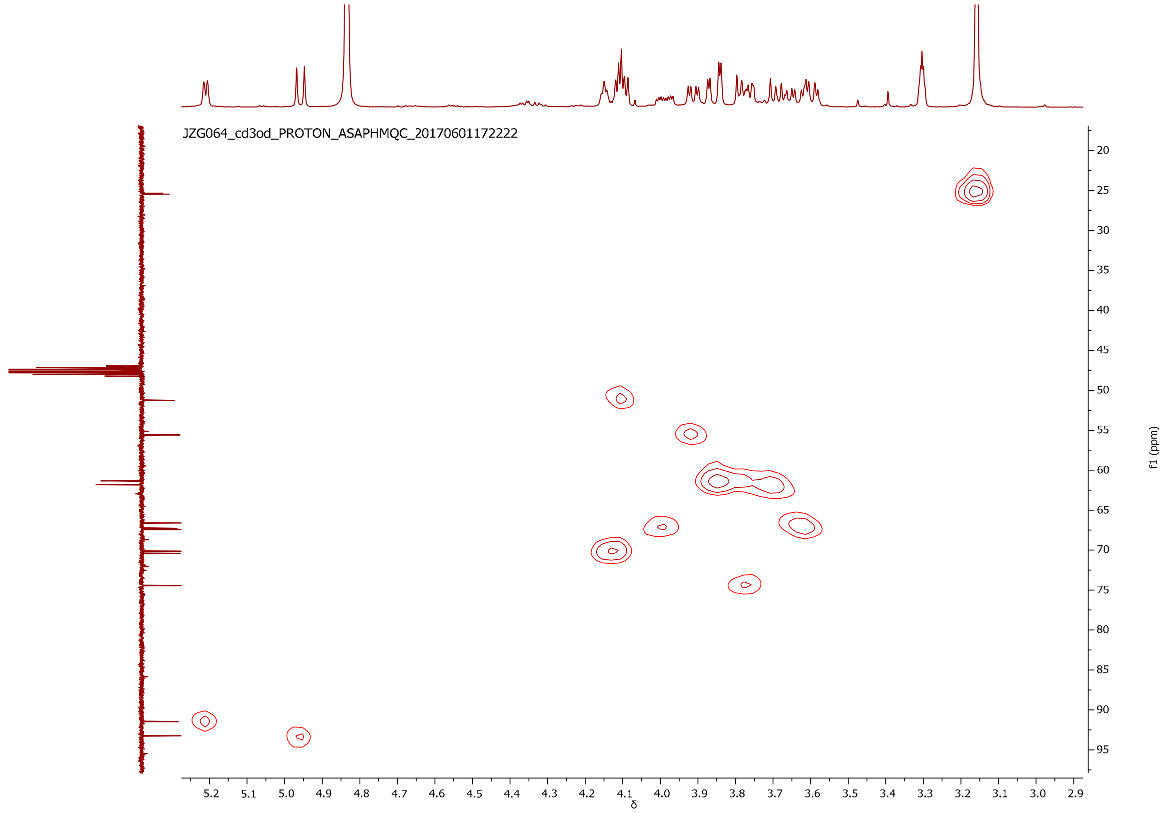
JZG064_cd3od_PROTON_ASAPHMQC_20170601172222
δ
f1 (ppm)

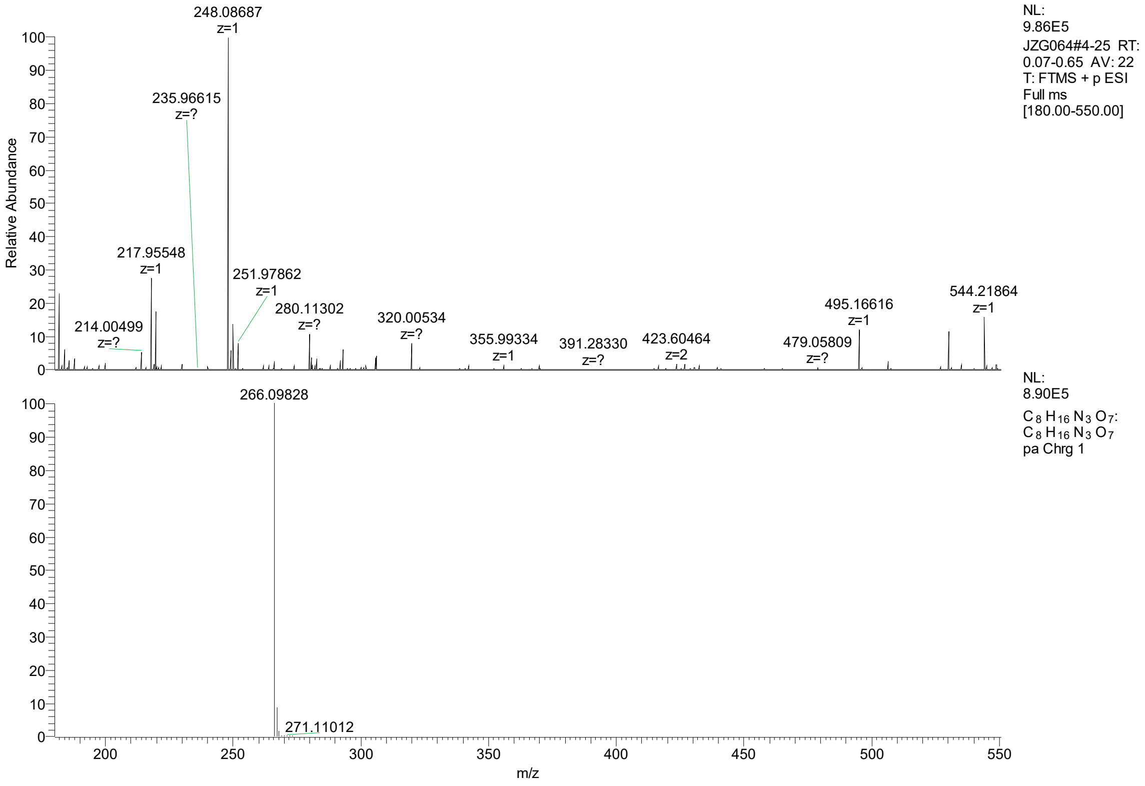
NL: 9.86E5
JZG064#4-25 RT: 0.07-0.65 AV: 22
T: FTMS + p ESI Full ms [180.00-550.00]
248.08687 z=1
235.96615 z=?
217.95548 z=1
251.97862 z=1
214.00499 z=?
280.11302 z=?
320.00534 z=?
355.99334 z=1
391.28330 z=?
423.60464 z=2
479.05809 z=?
495.16616 z=1
544.21864 z=1
NL: 8.90E5
C8 H16 N3 O7: C8 H16 N3 O7 pa Chrg 1
266.09828
271.11012
Relative Abundance
m/z

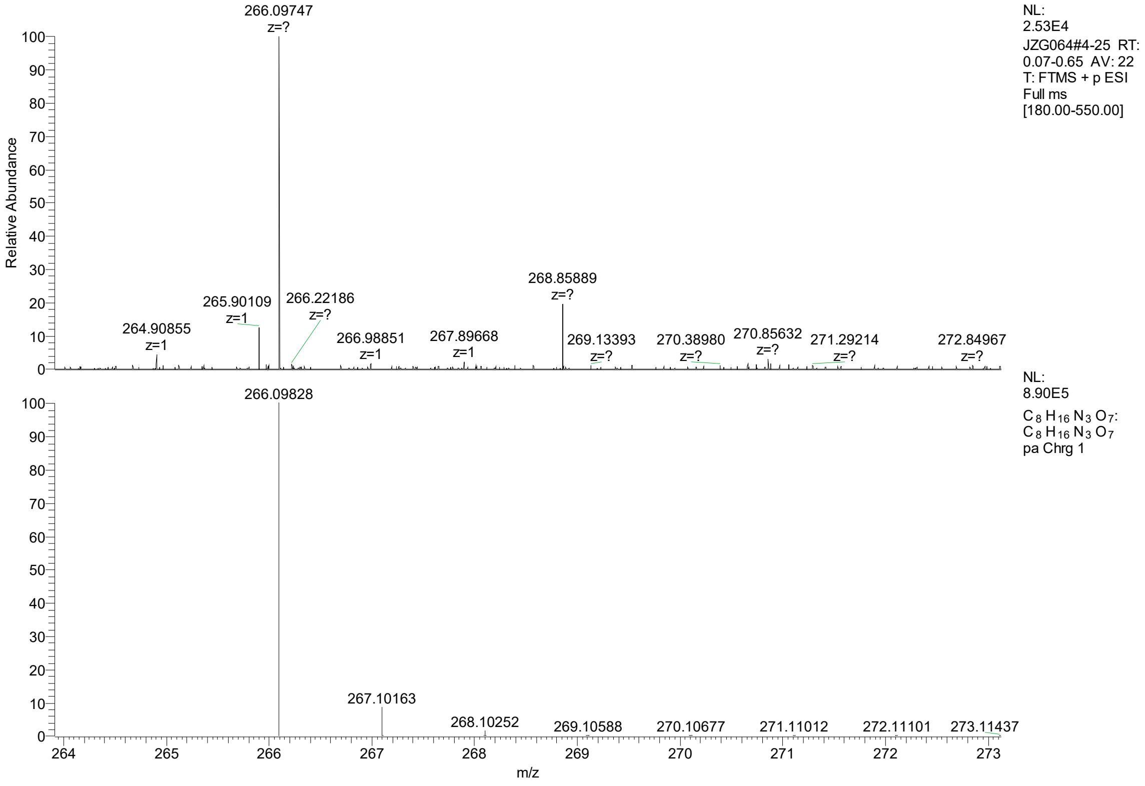
NL: 2.53E4
JZG064#4-25 RT: 0.07-0.65 AV: 22
T: FTMS + p ESI Full ms [180.00-550.00]
266.09747 z=?
264.90855 z=1
265.90109 z=1
266.22186 z=?
266.98851 z=1
267.89668 z=1
268.85889 z=?
269.13393 z=?
270.38980 z=?
270.85632 z=?
271.29214 z=?
272.84967 z=?
NL: 8.90E5
C8 H16 N3 O7: C8 H16 N3 O7 pa Chrg 1
266.09828
267.10163
268.10252
269.10588
270.10677
271.11012
272.11101
273.11437
Relative Abundance
m/z

PerkinElmer Spectrum Version 10.03.09
maandag 6 november 2017 18:44

Analyst Student
Date maandag 6 november 2017 18:44

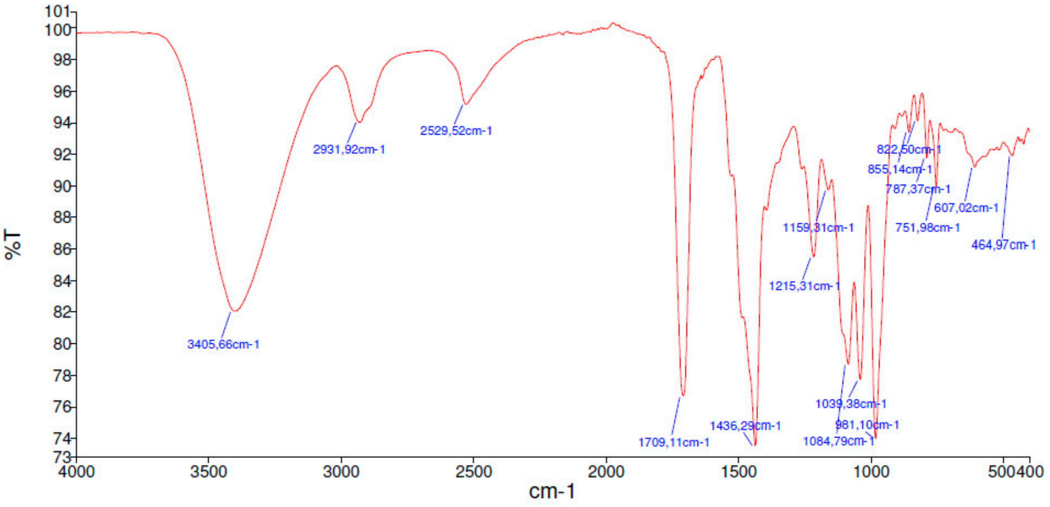

Student 11_013 Sample 011 By Student Date maandag, november 06 2017

Source Spectra Results									
Spectrum Name	Algorithm	ThresholdPercentT	ThresholdAbs	OtherUnits	PeakHeight	FindPeaks	FindBases	DisplayAbscissa	DisplayOrdinate
Student 11_013	Interpolated	0,5	0,0022	10	0,2	True	False	True	False

Page 1

Deoxy-streptozotocin (4)

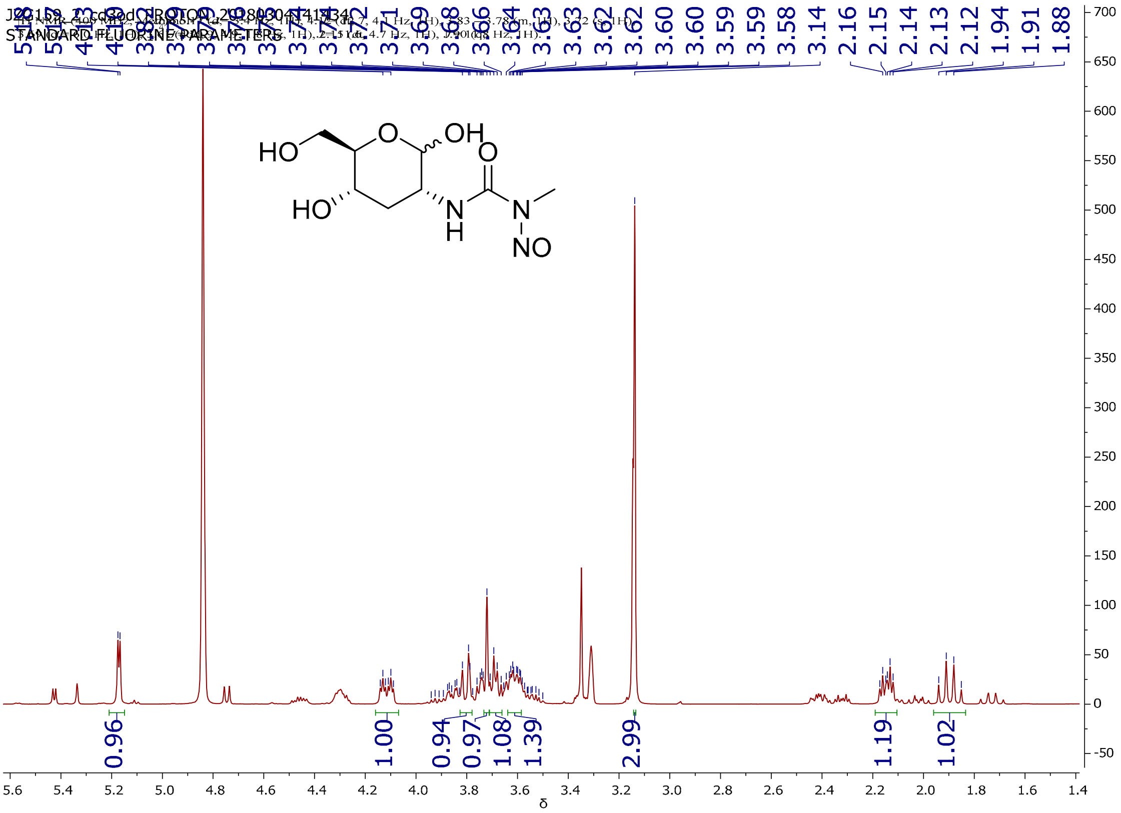

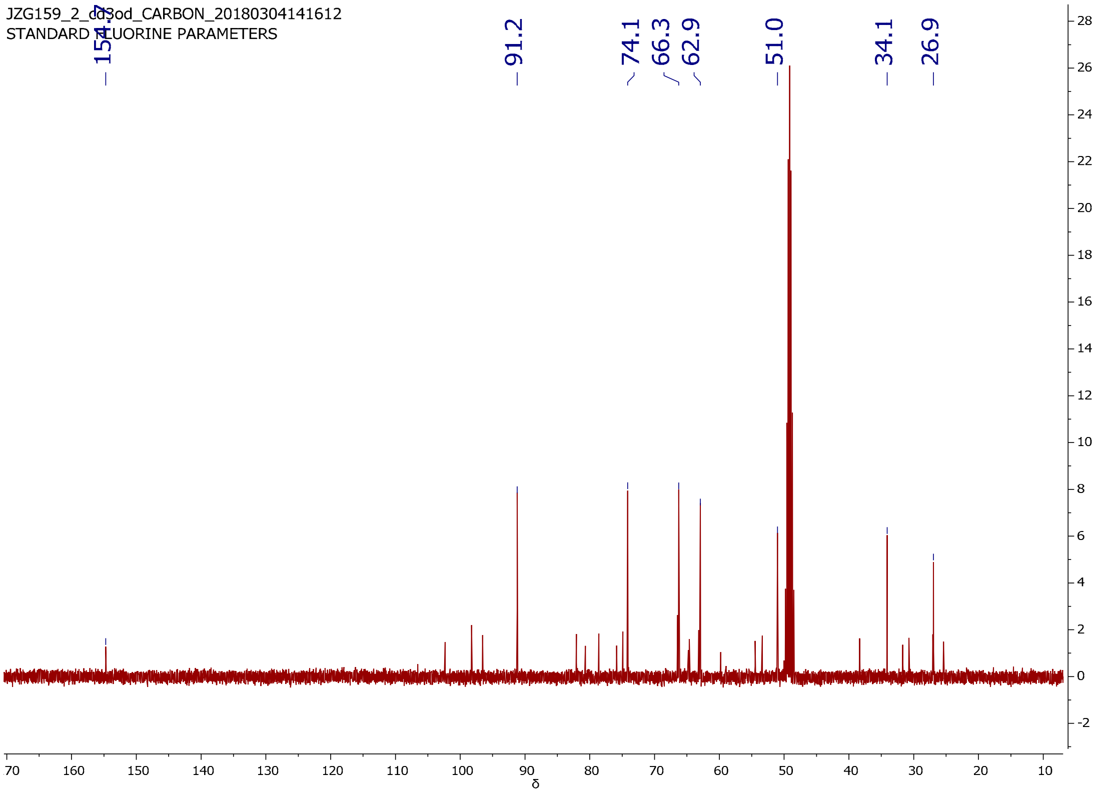

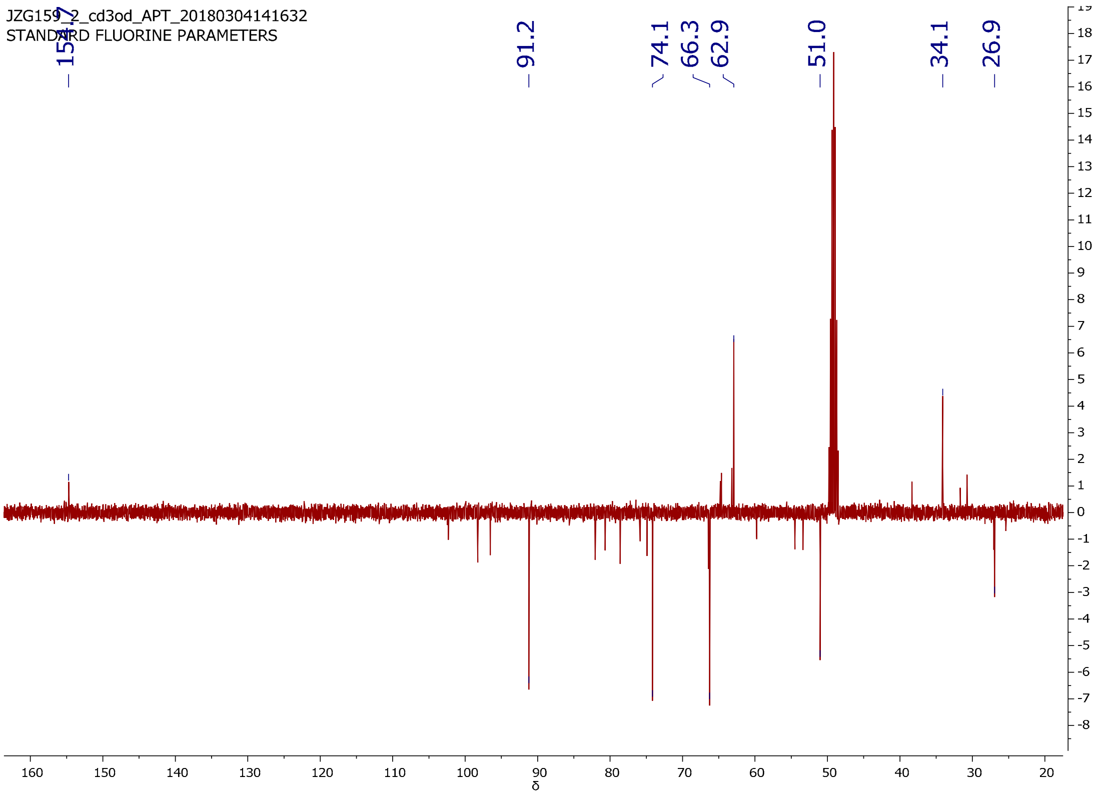

JZG159_2_cd3od_APT_20180304141632
STANDARD FLUORINE PARAMETERS
154.7
91.2
74.1
66.3
62.9
51.0
34.1
26.9
δ

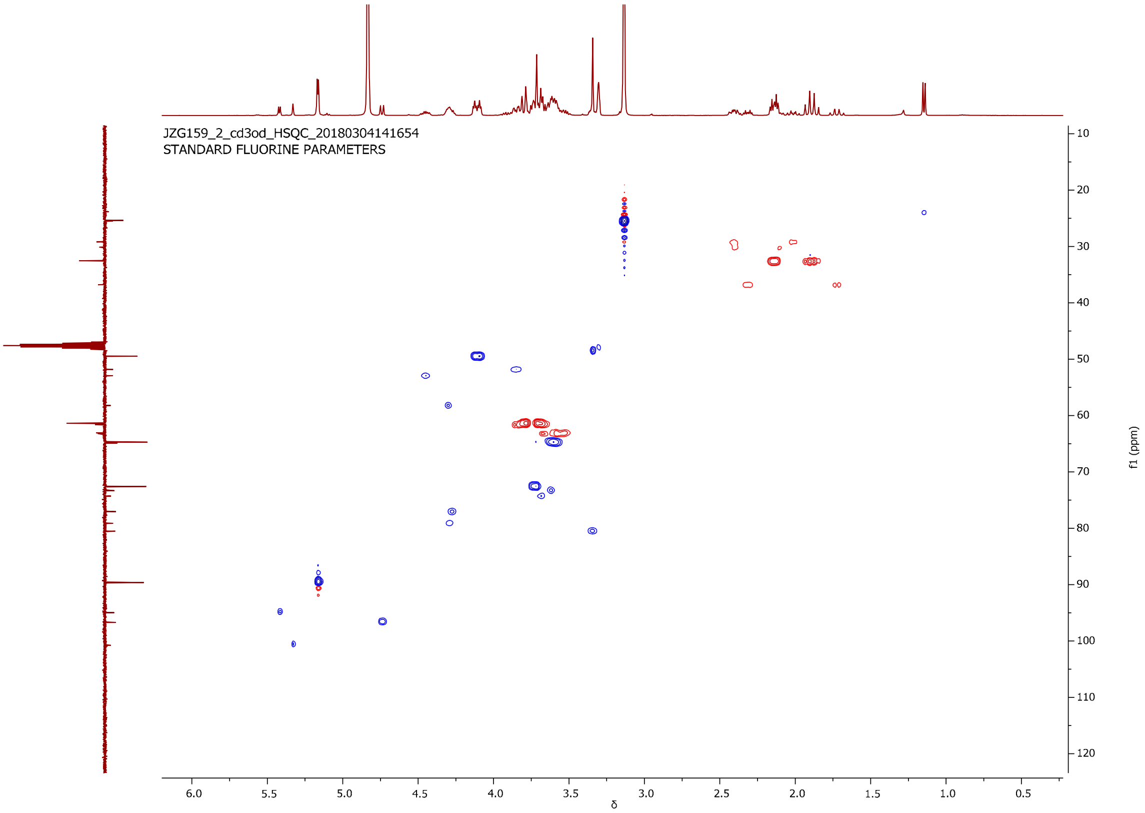

JZG159_2_cd3od_HSQC_20180304141654
STANDARD FLUORINE PARAMETERS
f1 (ppm)
δ

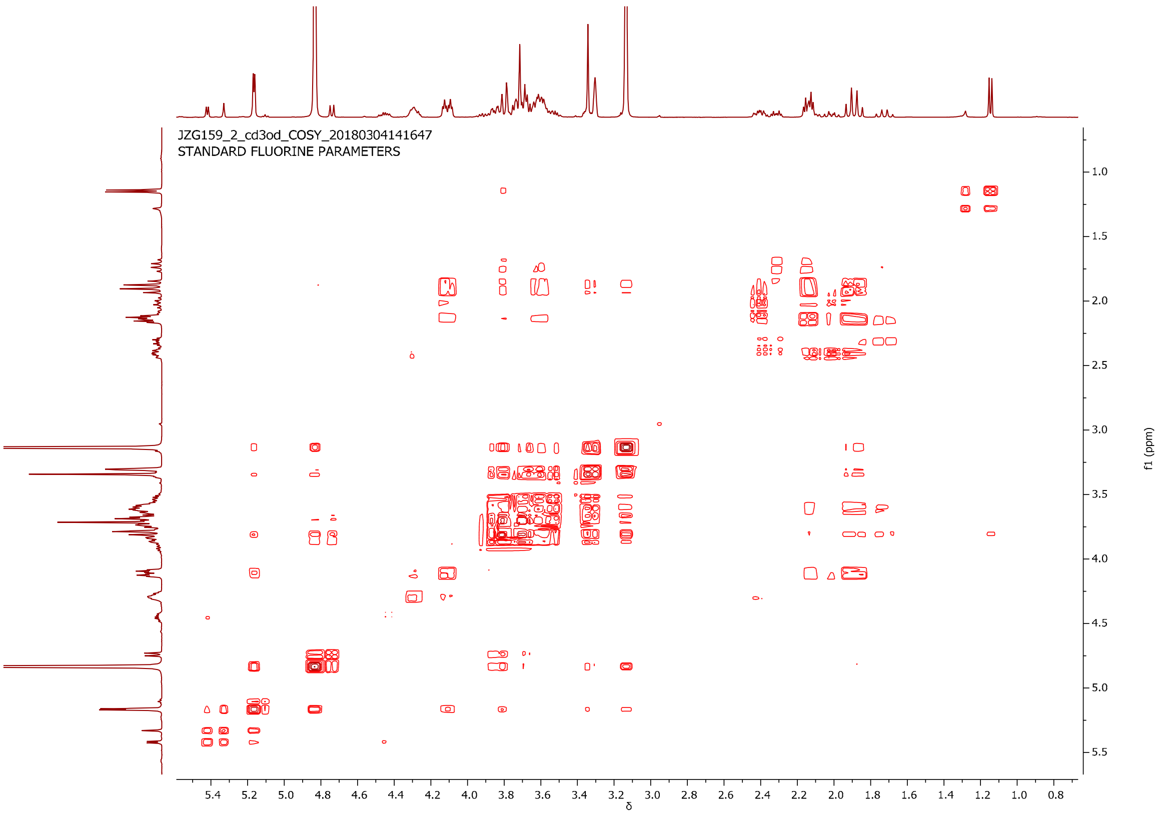
JZG159_2_cd3od_COSY_20180304141647
STANDARD FLUORINE PARAMETERS
f1 (ppm)
δ

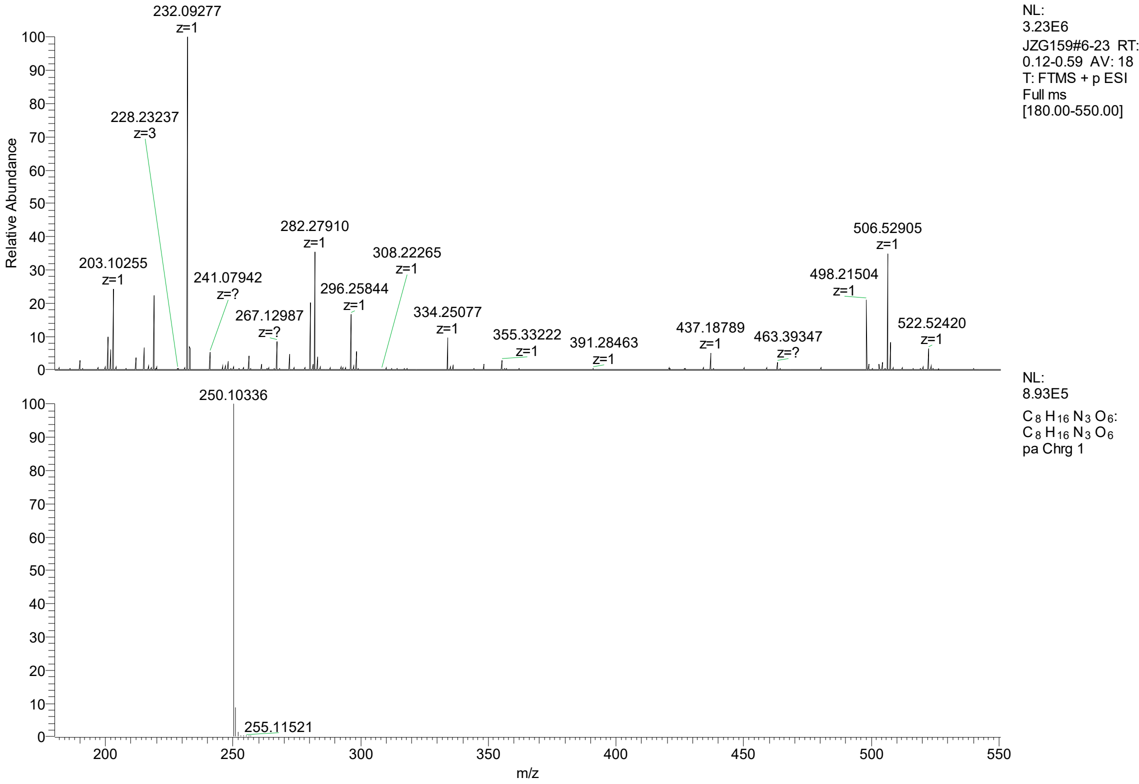
NL: 3.23E6
JZG159#6-23 RT: 0.12-0.59 AV: 18
T: FTMS + p ESI Full ms [180.00-550.00]
Relative Abundance
232.09277 z=1
228.23237 z=3
203.10255 z=1
241.07942 z=?
267.12987 z=?
282.27910 z=1
296.25844 z=1
308.22265 z=1
334.25077 z=1
355.33222 z=1
391.28463 z=1
437.18789 z=1
463.39347 z=?
498.21504 z=1
506.52905 z=1
522.52420 z=1
NL: 8.93E5
C8 H16 N3 O6: C8 H16 N3 O6 pa Chrg 1
250.10336
255.11521
m/z

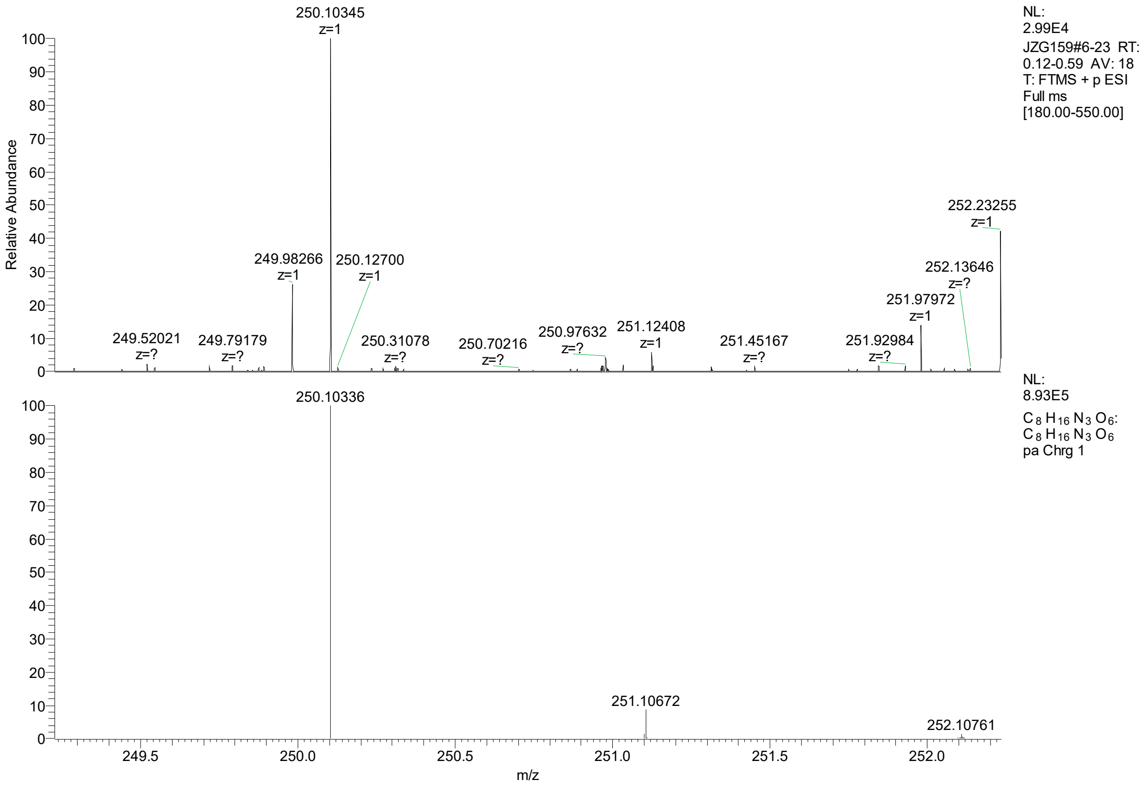

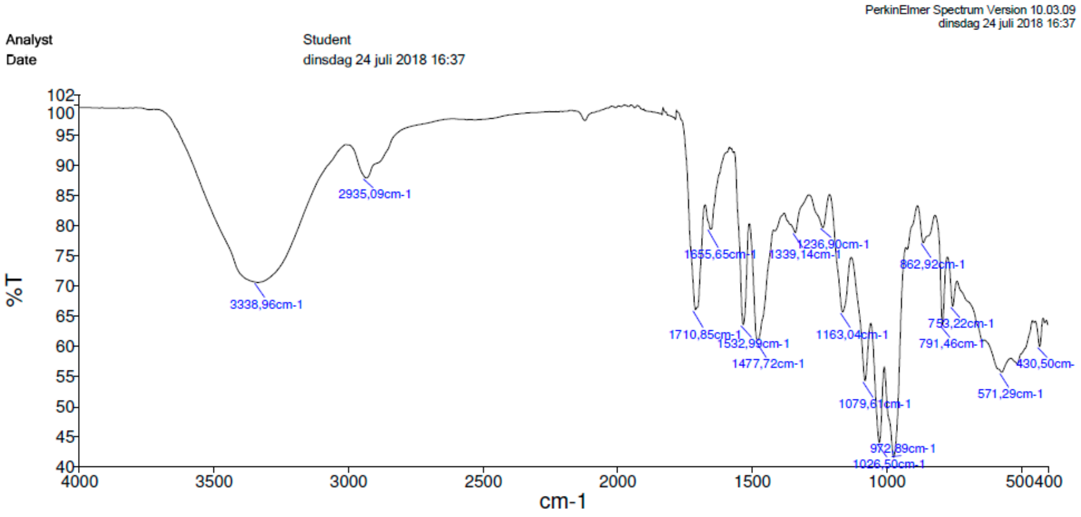

Sample Name	Description	Quality Checks
Student 01_039	Sample 001 By Student Date dinsdag, juli 24 2018	The Quality Checks do not report any warnings for the sample.

N-methyl 4-nitrophenyl carbamate (5)

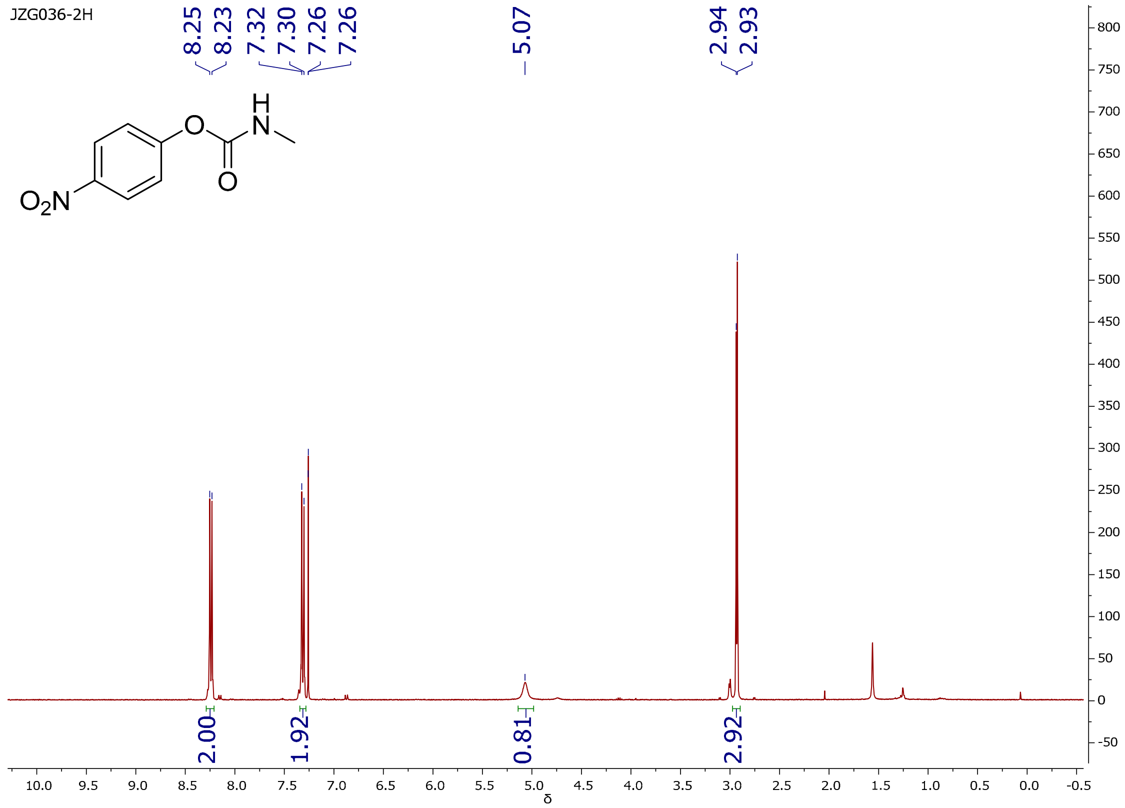

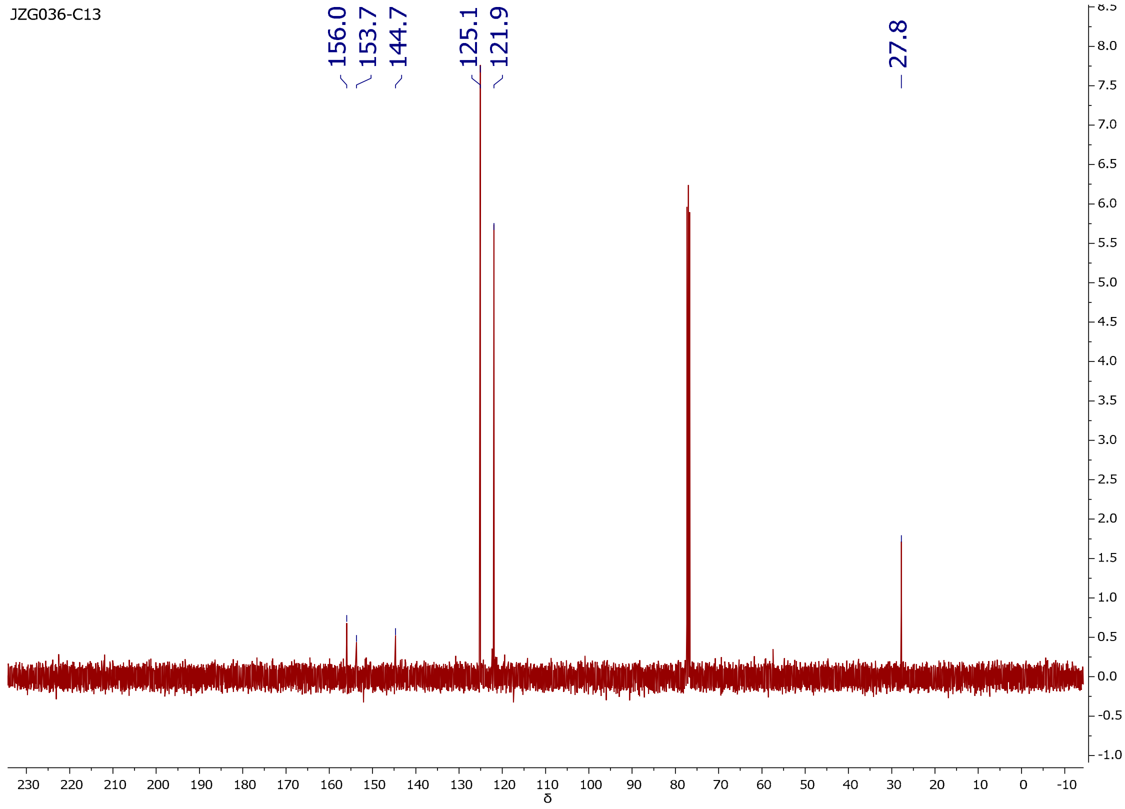

4-nitrophenyl *N*-nitroso-*N*-methylcarbamate (6)

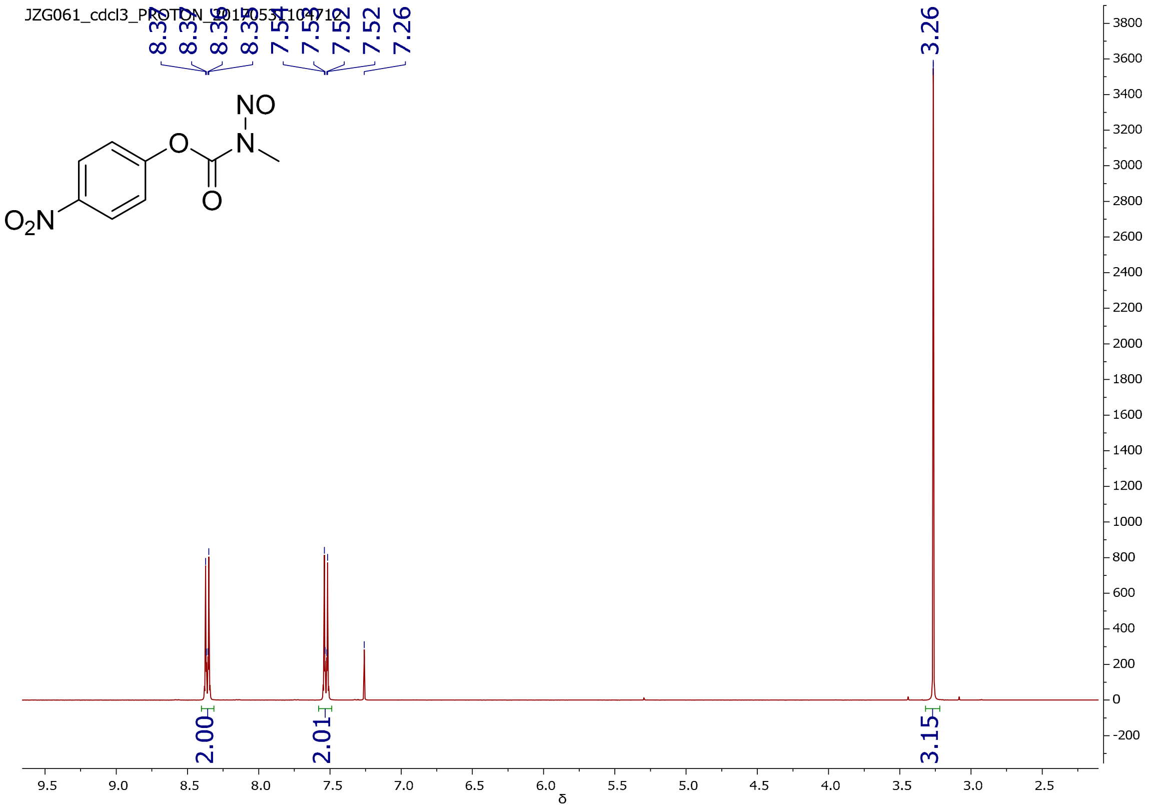

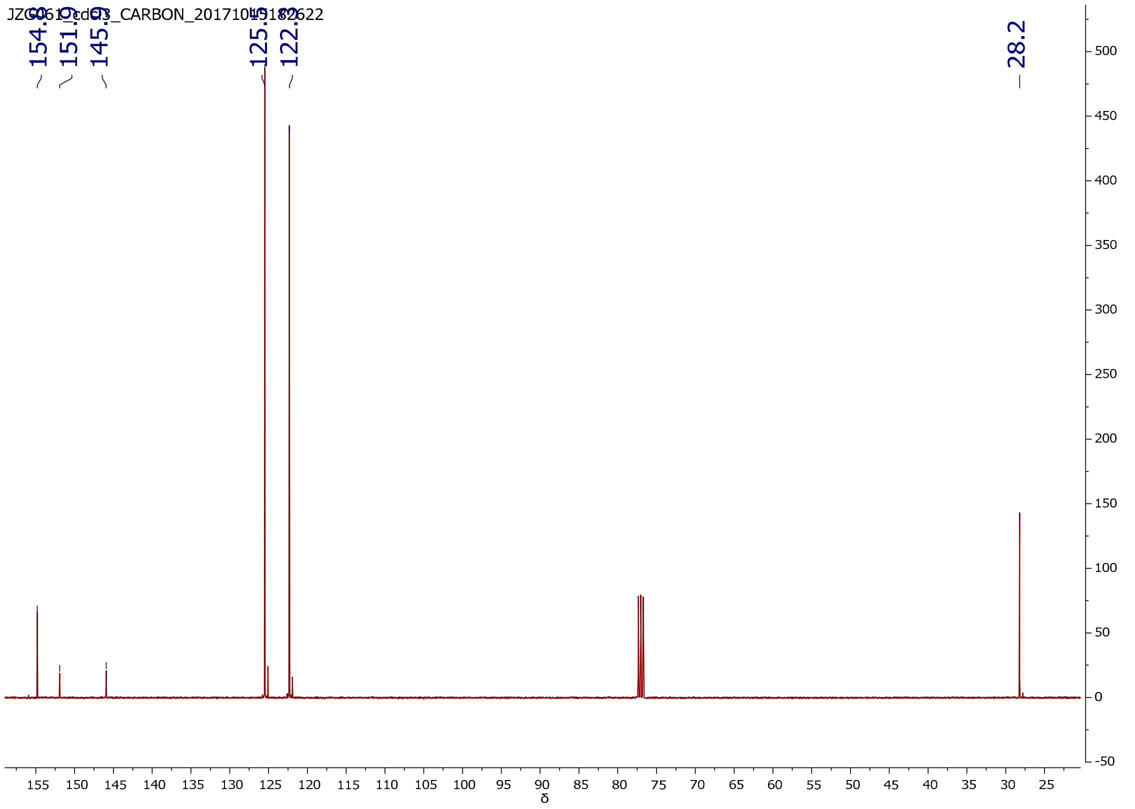

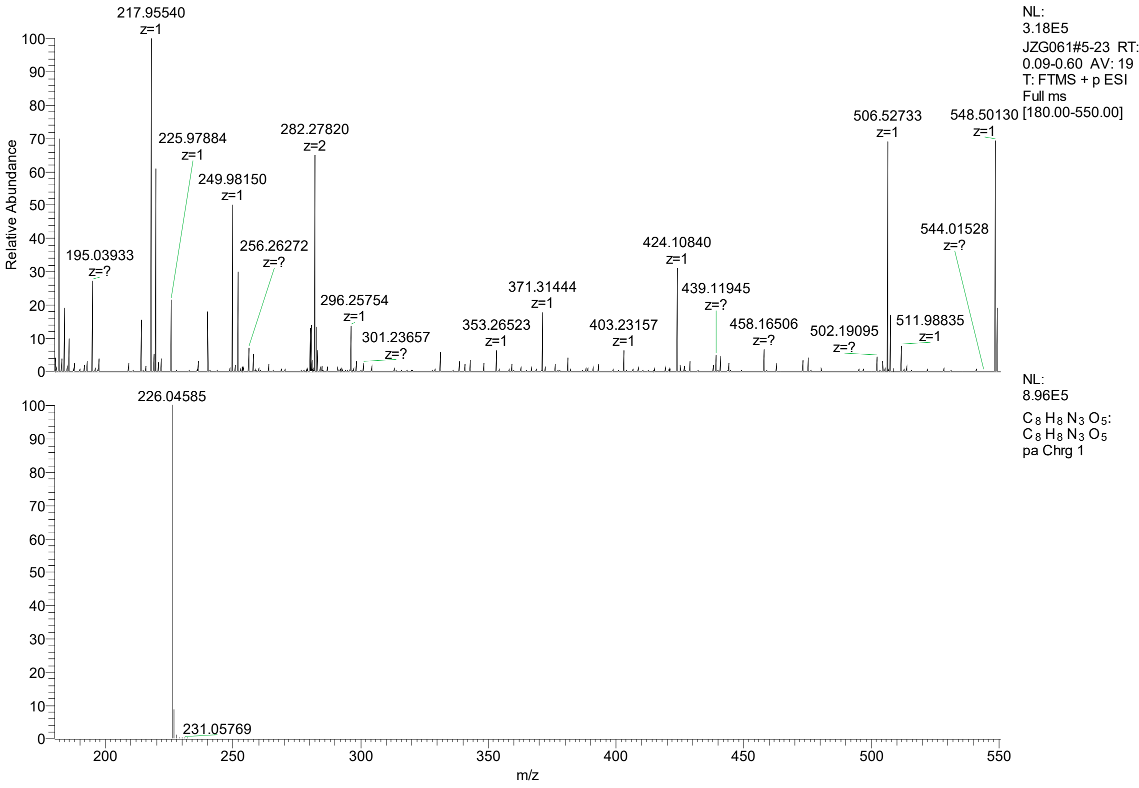

NL: 3.18E5
JZG061#5-23 RT: 0.09-0.60 AV: 19
T: FTMS + p ESI Full ms [180.00-550.00]
NL: 8.96E5
C8 H8 N3 O5: C8 H8 N3 O5 pa Chrg 1
217.95540 z=1
225.97884 z=1
249.98150 z=1
282.27820 z=2
195.03933 z=?
256.26272 z=?
296.25754 z=1
301.23657 z=?
353.26523 z=1
371.31444 z=1
403.23157 z=1
424.10840 z=1
439.11945 z=?
458.16506 z=?
502.19095 z=?
506.52733 z=1
511.98835 z=1
544.01528 z=?
548.50130 z=1
226.04585
231.05769
Relative Abundance
m/z

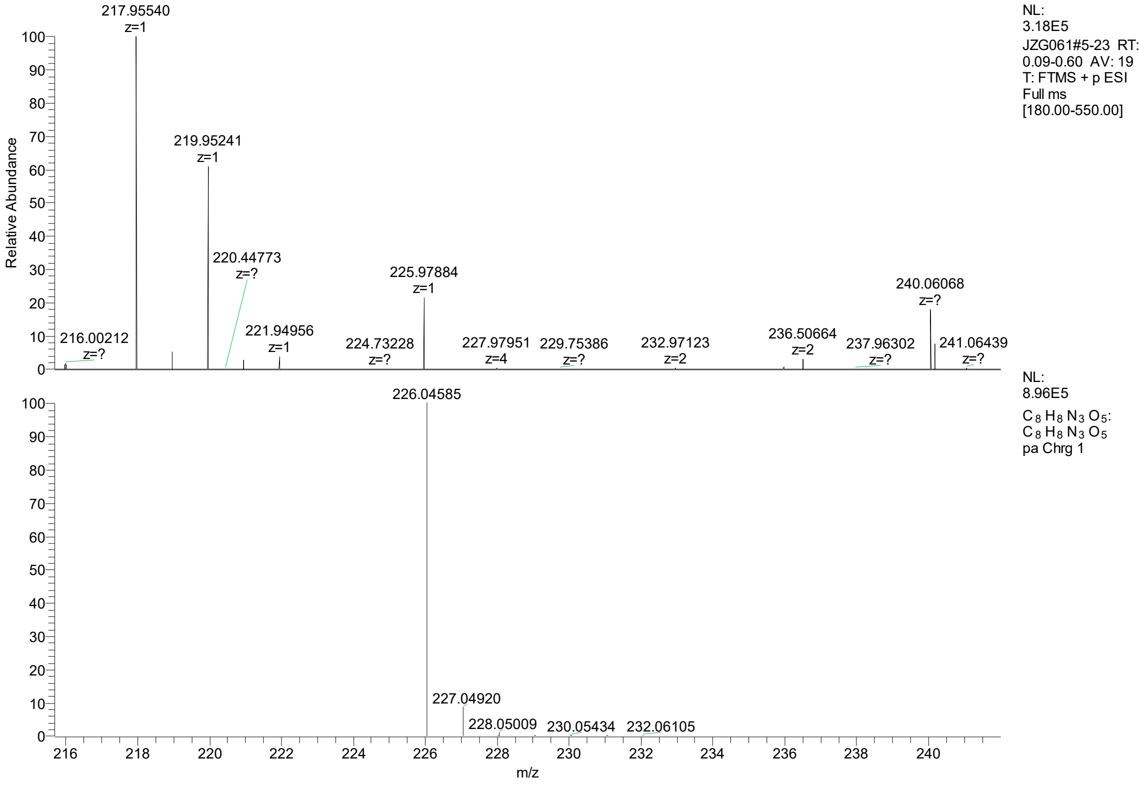

NL: 3.18E5
JZG061#5-23 RT: 0.09-0.60 AV: 19
T: FTMS + p ESI Full ms [180.00-550.00]
NL: 8.96E5
C8 H8 N3 O5: C8 H8 N3 O5 pa Chrg 1
217.95540 z=1
219.95241 z=1
220.44773 z=?
216.00212 z=?
221.94956 z=1
224.73228 z=?
225.97884 z=1
227.97951 z=4
229.75386 z=?
232.97123 z=2
236.50664 z=2
237.96302 z=?
240.06068 z=?
241.06439 z=?
226.04585
227.04920
228.05009
230.05434
232.06105
Relative Abundance
m/z

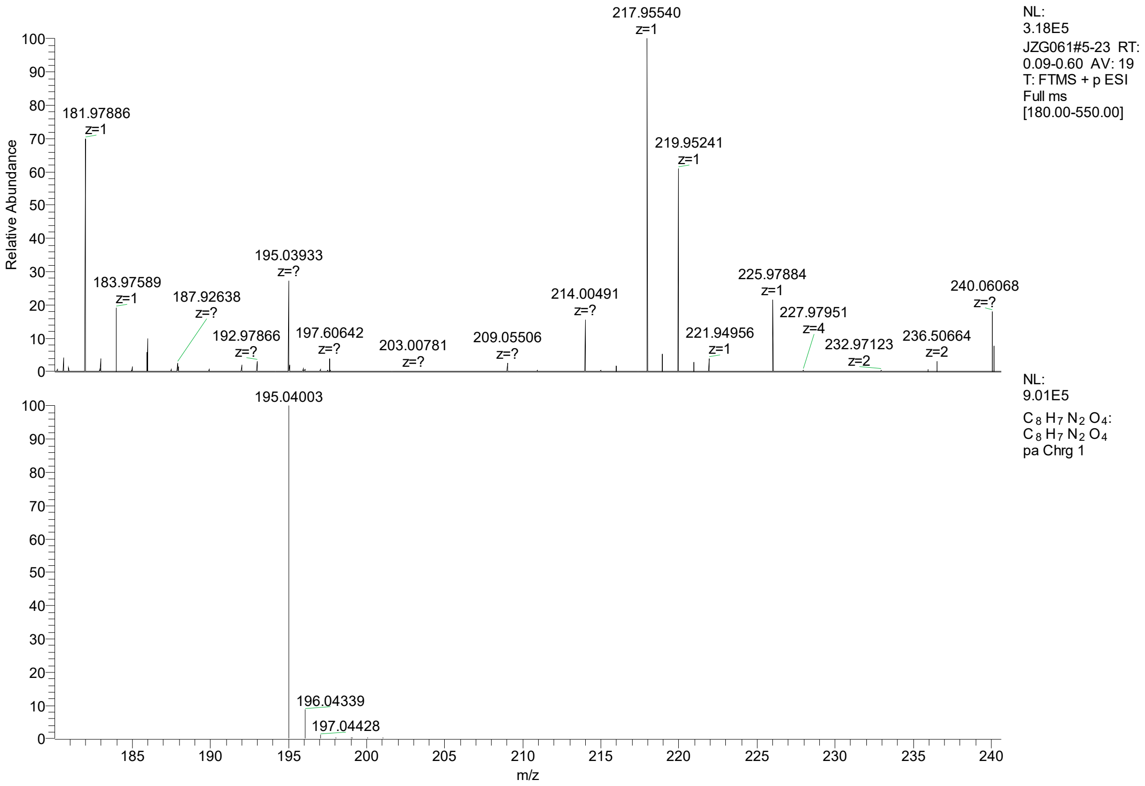
NL: 3.18E5
JZG061#5-23 RT: 0.09-0.60 AV: 19
T: FTMS + p ESI Full ms [180.00-550.00]
Relative Abundance
217.95540 z=1
181.97886 z=1
219.95241 z=1
195.03933 z=?
183.97589 z=1
187.92638 z=?
225.97884 z=1
240.06068 z=?
214.00491 z=?
192.97866 z=?
197.60642 z=?
203.00781 z=?
209.05506 z=?
221.94956 z=1
227.97951 z=4
232.97123 z=2
236.50664 z=2
NL: 9.01E5
C8 H7 N2 O4: C8 H7 N2 O4 pa Chrg 1
195.04003
196.04339
197.04428
m/z

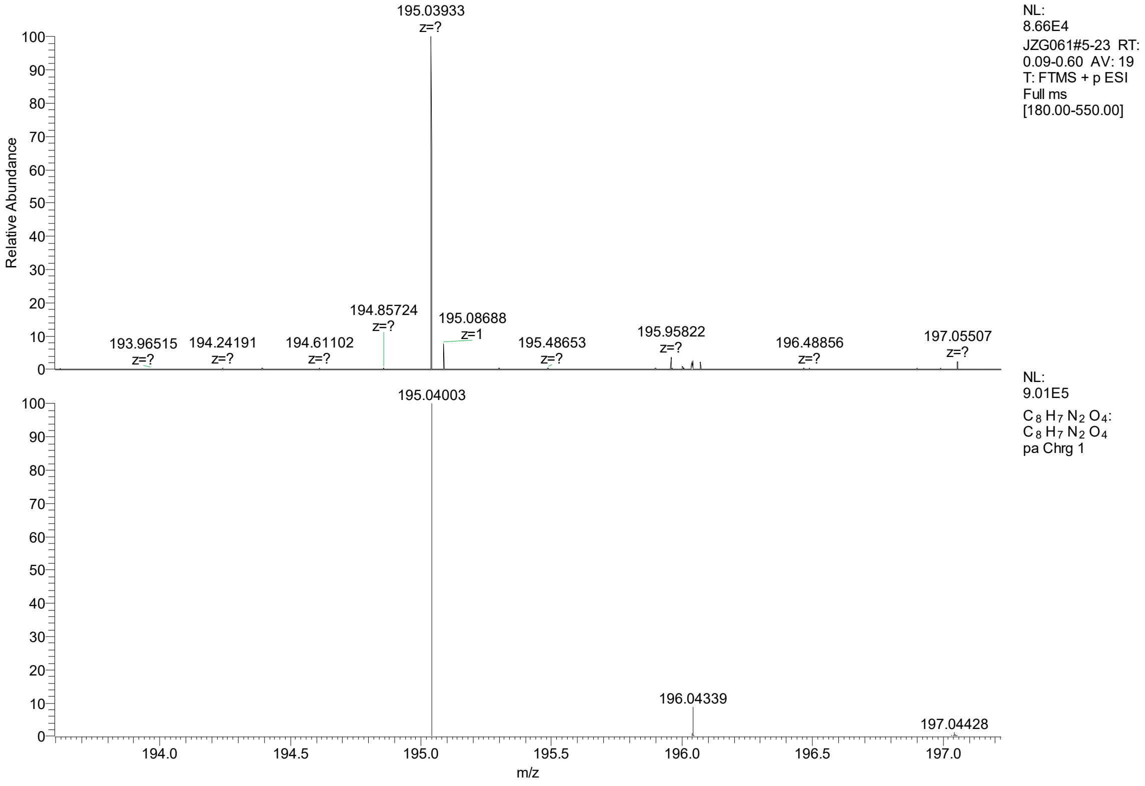
NL: 8.66E4
JZG061#5-23 RT: 0.09-0.60 AV: 19
T: FTMS + p ESI Full ms [180.00-550.00]
Relative Abundance
195.03933 z=?
194.85724 z=?
195.08688 z=1
193.96515 z=?
194.24191 z=?
194.61102 z=?
195.48653 z=?
195.95822 z=?
196.48856 z=?
197.05507 z=?
NL: 9.01E5
C8 H7 N2 O4: C8 H7 N2 O4 pa Chrg 1
195.04003
196.04339
197.04428
m/z

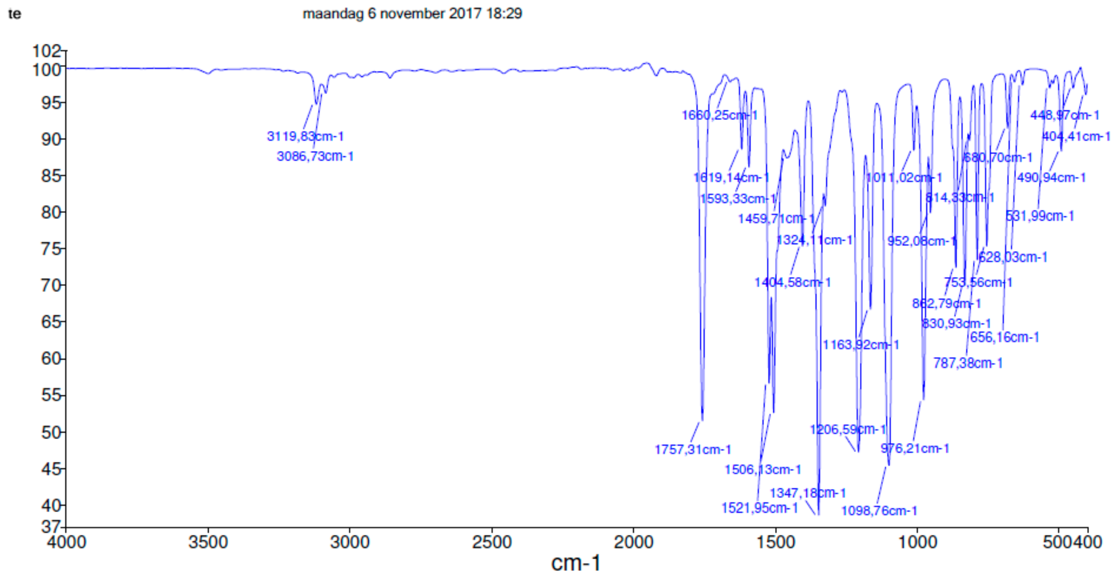
te
maandag 6 november 2017 18:29
3119,83cm-1
3086,73cm-1
1660,25cm-1
1619,14cm-1
1593,33cm-1
1459,71cm-1
1324,11cm-1
1404,58cm-1
1757,31cm-1
1506,13cm-1
1521,95cm-1
1347,18cm-1
1206,59cm-1
1163,92cm-1
1098,76cm-1
1011,02cm-1
976,21cm-1
952,08cm-1
914,33cm-1
862,79cm-1
830,93cm-1
787,38cm-1
753,56cm-1
680,70cm-1
656,16cm-1
628,03cm-1
531,99cm-1
490,94cm-1
448,97cm-1
404,41cm-1
cm-1
